# Applications of Raman spectroscopy for microplastic detection and characterization: a comprehensive spectral reference

**DOI:** 10.1007/s11356-025-37224-3

**Published:** 2025-11-27

**Authors:** Yasemin Umurhan, Mackenzie Songsart-Power, Tej B. Limbu, Tuan Phan

**Affiliations:** 1 Department of Environmental and Interdisciplinary Science, Texas Southern University, Houston, TX USA; 2 Department of Physical and Applied Sciences, University of Houston–Clear Lake, Houston, TX USA; 3 Department of Chemistry, Texas Southern University, Houston, TX USA

**Keywords:** Raman spectroscopy, Microplastics detection, Polymer identification, Environmental contamination, Spectral library

## Abstract

Microplastics (MPs), plastic particles smaller than 5 mm, represent an escalating global concern due to their persistence, ubiquity, and potential risks to ecosystems and human health. This review critically examines the application of Raman spectroscopy as a possibly non-destructive vibrational technique for detecting and characterizing MPs in environmental and biological matrices. The main objective is to consolidate Raman spectral signatures of common polymers such as polystyrene, polyester, and polyethylene terephthalate and to evaluate methodological advances that improve analytical precision and detection sensitivity. By summarizing Raman-based approaches across water, sediment, air, and biological tissues, this review identifies major analytical challenges including fluorescence interference and matrix complexity and discusses recent innovations such as coherent anti-Stokes Raman spectroscopy (CARS), surface-enhanced Raman spectroscopy (SERS), and compressive Raman technology (CRT). Overall, this work provides a comprehensive reference for Raman spectral data and offers practical insights to guide future research aimed at advancing MP detection and pollution mitigation.

**Supplementary Information:**

The online version contains supplementary material available at 10.1007/s11356-025-37224-3.

## Introduction

### Historical development and ubiquity of plastics

Since the invention of Bakelite in the early twentieth century, plastics, valued for their ease of production, affordability, durability, and versatility, have become indispensable in modern life (Feldman 2008). In the following decades, many synthetic polymers were developed, including polyesters (PES), poly(vinyl chloride) (PVC), polystyrene (PS), polyacrylonitrile (PAN), polymethyl methacrylate (PMMA), and polyamides (PA), such as nylon (Feldman 2008; Burford 2019; Evode et al. 2021). The discovery of polytetrafluoroethylene (PTFE) in 1938; the large-scale production of polyethylene (PE) in the 1950s; the synthesis of poly(ethylene terephthalate) (PET) in 1955 for use in plastics, films, and fibers; and the introduction of polypropylene (PP) in 1957 further expanded the range of plastics and laid the foundation for widespread industrial and consumer applications (Feldman 2008). However, their persistence has led to significant environmental accumulation, and approximately 79% of 6300 million tonnes of mismanaged plastic waste on land typically end up in rivers and lakes, making these freshwater systems the primary sources of plastic pollution in the oceans (Azevedo-Santos et al. 2021; Bodor et al. 2024). It has been estimated that there are 5.25 trillion pieces of plastic (270,000 tonnes) in the oceans and that plastic particles carried by wind and atmospheric transport travel globally, accumulating and returning to both land and water (Araujo et al. 2018; Feldman 2008; Gaston et al. 2020; Jin et al. 2022; O'Brien et al. 2023; Phan et al. 2022; Schymanski et al. 2017).

### Definition, sources, and environmental distribution of MPs

MPs, defined as particles from 1 µm to 5 mm, arise from primary sources (e.g., microbeads in cosmetics) or secondary degradation of larger plastics due to sunlight, mechanical forces, and biological processes (Liu et al. 2022; Mikac et al. 2023). The degradation of plastics, typically caused by exposure to UV radiation, mechanical stress from wind and water waves, temperature fluctuations, hydrolysis, and enzymatic activity of microorganisms, is an effective mechanism for the formation of secondary MPs (Torres-Agulló et al. 2021). MPs infiltrate terrestrial, aquatic, and atmospheric environments, entering food chains and biological tissues, including human blood, breast milk, and organs, posing ecological and health risks (Ribeiro-Claro et al. 2017; Mikac et al. 2023; Miserli et al. 2023; Liu M et al*.* 2023; Genchi et al. 2023; Massardo et al. 2024).

### Ecological and human health implications

MPs can damage tissues and organs in organisms, affecting the gastrointestinal, respiratory, endocrine, reproductive, and immune systems by disrupting gut microbiota and serving as carriers for toxic chemicals and pathogens (Lee Y et al. 2023; Mariano et al. 2021; Xu et al. 2019). They can accumulate in aquatic organisms and commercial seafood, such as mussels and shellfish, and be transmitted to humans through diet (Lee Y et al. 2023; Xu et al. 2019), raising concerns about long-term toxicity and potential neurotoxic effects (Lee Y et al. 2023; Manolaki et al. 2023; Xu et al. 2019). Furthermore, chemical additives in plastics, including bisphenol A, phthalates, and flame retardants, can disrupt hormonal functions and cause developmental and reproductive disorders (Lee Y et al. 2023; Troost et al. 2024).

### Objective and scope of this review

Accurate detection of MPs is critical to assess their distribution and impacts. Raman spectroscopy, based on inelastic light scattering, provides molecular fingerprints for precise polymer identification (Ribeiro-Claro et al. 2017). This review aims to provide a comprehensive reference for Raman spectral signatures of MPs, synthesize analytical advancements, and guide future research to address MP pollution.

### Literature search and review methodology

This review was conducted systematically to consolidate Raman-based approaches for MP detection and characterization, ensuring comprehensive coverage and reproducibility. In total, the review incorporates 207 references. Literature searches were performed across major academic databases: Web of Science, Scopus, PubMed, and Google Scholar. Searches were conducted iteratively between January 2024 and October 2025 with no formal start date to capture foundational works while prioritizing post-2010 publications on MPs.

Search strings combined core terms: (“Raman spectroscopy” OR “Raman microspectroscopy” OR “CARS” OR “SERS”) AND (“microplastic*” OR “microplastics” OR “nanoplastic*” OR “plastic debris” OR “polymer particles”) AND (“detection” OR “identification” OR “characterization” OR “spectral library” OR “environmental matrix” OR “biological sample”). Filters included English-language peer-reviewed articles, reviews, and book chapters; exclusions applied to non-Raman techniques (pure Fourier Transform Infrared Spectroscopy (FTIR) studies) or unrelated fields (medical Raman without MP context).

Inclusion criteria encompassed studies reporting Raman spectral data for common MPs (in environmental/biological matrices; methodological details on sample preparation, detection limits, or innovations; and empirical or review articles with ≥ 1 Raman application to MPs). Exclusions covered non-empirical works (opinion pieces), studies lacking spectral profiles or Raman-specific outcomes, duplicates, or preprints without peer review.

## Sample preparation methods for Raman spectroscopy of MPs

Quality assurance and control (QA/QC) are essential to ensure the sensitivity and accuracy of Raman spectroscopy analysis of MPs. To minimize contamination during sample collection and preparation, researchers should wear natural fiber clothing and cotton lab coats and avoid using plastic materials in both field and laboratory settings (Gaston et al. 2020; Ribeiro-Claro et al. 2017). All sampling containers, filters, glassware, and tweezers should be rinsed three times with deionized water before use (Gaston et al. 2020; Ribeiro-Claro et al. 2017). Procedural controls are also necessary to monitor potential contamination from equipment, reagents, and the laboratory environment (Ribeiro-Claro et al. 2017).

Table 1 summarizes the sample preparation methods used for Raman spectroscopy analysis of MPs. Sample preparation generally involves four main steps: (a) collection and isolation; (b) shredding, separation, filtration, and drying; (c) visual and Raman spectral analysis; and (d) MP identification. However, protocols vary depending on sample type, with their efficiency largely influenced by the amount of organic or inorganic residues, polymer type, and target particle size.

**Table 1:** Sample preparation methods for Raman spectroscopy of MPs

Sample type	Preparation step	Digestion method	Filtration method	Storage method	Ref
Water	Filter through gold-plated polycarbonate or stainless-steel filters (pore size ~ 1.2 µm); rinse and air dry	Typically, not required unless contaminated	Gold-plated polycarbonate or stainless-steel filters	Petri dishes (with lids) or covered with aluminum foil	Genchi et al. (2023); Schymanski et al. (2017)
Air	Collect directly onto filters; rinse with deionized water	Apply digestion to remove organics if needed; Nile Red staining used for MP quantification	Same as above	Same as above	Gaston et al. (2020)
Sediment	Density separation using NaI, NaCl, ZnCl₂ (MP density: 0.8–1.4 g/cm^3^), or canola oil	30% H₂O₂ if necessary; 30% KOH and NaClO in a ratio of 1:1	Same as above	Same as above	Rani et al. (2023); Ribeiro-Claro et al. (2017)
Biological	Remove soft tissues (if present); rinse sample	*Alkaline*: 10% KOH at 60 °C for 24 h (preferred for tissues); *Oxidative*: Fenton’s reagent (0.05 M FeSO₄·7H₂O + 30% H₂O₂, 1:1 v/v) at pH 3, 25 °C; *Enzymatic*: proteinase K (minimal MP impact; slower, incomplete)	Filter with appropriate pore	Same as above	Araujo et al. (2018); Massardo et al. (2024); Miserli et al. (2023); Ribeiro-Claro et al. (2017)
Quality assurance/quality control	Wear natural fiber clothing and cotton lab coats; rinse all containers and tools with DI water × 3; avoid plastics	Use procedural blanks to monitor contamination	Use non-plastic materials for filtering	Maintain sample integrity by avoiding exposure	Ribeiro-Claro et al. (2017); Gaston et al. (2020)

Water samples are commonly filtered through gold-plated polycarbonate, glass microfiber, or stainless-steel filters with pore sizes ranging from 1.2 to 3 µm to retain smaller MPs (Genchi et al. 2023; Kwon et al. 2022; Schymanski et al*.* 2017). After filtration, samples are rinsed with distilled water, air-dried, and stored in covered Petri dishes or under aluminum foil until analysis (Genchi et al. 2023; Schymanski et al*.* 2017). Recently, silicon (Si) filters and Si nanomembranes have been introduced to enhance MP detection, particularly for the smallest size fractions (Madejski et al. 2020; Romanick et al. 2025). These filters provide a clean substrate suitable for direct Raman measurements, are chemically inert to digestion reagents such as 4 M KOH and 30% H₂O₂, and generate low background signals during Raman and other imaging analyses. Despite these advantages, Si filters are expensive, and the Si peak in Raman spectra can overlap with polymer signals, limiting broader application.

Air samples are generally collected directly onto filters to capture airborne MPs (Gaston et al. 2020). Trapped particles are rinsed with deionized water, followed by digestion to remove organic matter. Nile Red staining is frequently used to visualize and quantify MPs, although differences in dye affinity among polymers can introduce variability (Gaston et al. 2020). Raman analysis conducted after digestion provides higher reliability, but current procedures vary widely. Standardizing digestion steps and reducing reliance on staining alone are recommended to improve reproducibility (Prata et al. 2021).

Sediment samples, which often contain complex organic and inorganic matrices, typically undergo density separation to isolate MPs with densities between 0.8 and 1.4 g/cm^3^ (Ribeiro-Claro et al. 2017). Separation can be achieved using solutions such as NaCl, NaI, or ZnCl₂, while the buoyancy of oil has also been exploited for soil or sediment samples (Sheriff et al. 2024; Zhao et al. 2024). More concentrated liquids such as lithium metatungstate (LMT, 1.62 g/mL) and sodium polytungstate (SPT, 1.0–3.1 g/cm^3^ depending on concentration) have proven effective for density-based separation (Sheriff et al. 2024; Uguagliati et al. 2023). ZnCl₂ provides the most efficient MP recovery due to its high density, but its cost and potential toxicity are significant limitations. Reporting density solution parameters remains critical for cross-study comparability. Digestion is generally avoided in sediment analysis to prevent MP degradation, although mild alkaline pretreatment with KOH can be used for highly organic sediments (Ribeiro-Claro et al. 2017).

Biological samples are among the most challenging matrices for MP analysis due to their high organic content, which can obscure Raman signals. Effective digestion is therefore essential to remove biological residues while preserving MPs. Alkaline digestion using 10% KOH at 60 °C for 24 h efficiently dissolves tissues without degrading MPs (Araujo et al. 2018; Massardo et al. 2024; Ribeiro-Claro et al. 2017). In comparative studies on mussels, crabs, and fish tissues, KOH digestion yielded favorable recovery of MPs compared to oxidative methods (Araujo et al. 2018; Ribeiro-Claro et al. 2017). NaOH, however, should be avoided since it can degrade PET particles (Ribeiro-Claro et al. 2017).

Oxidative digestion using Fenton’s reagent (Fe^2^⁺/H₂O₂) is another effective approach for biological and soil matrices (Miserli et al. 2023; Rani et al. 2023; Schrank et al. 2022; Thomas et al. 2020). This reagent is typically prepared by mixing 0.05 M FeSO₄·7H₂O with 30% H₂O₂ in a 1:1 volume ratio, adjusted to pH 3 with concentrated H₂SO₄, and maintained at 25 °C in glass containers (Miserli et al. 2023). Following digestion, MPs are separated by density flotation using a 2 M ZnCl₂ solution, mixed for 30 min, and left to settle before recovery through centrifugation and vacuum filtration (Miserli et al. 2023).

Enzymatic digestion using proteinase K offers a gentle alternative for degrading organic matter with minimal impact on MP integrity (Araujo et al. 2018). However, the process is slow, costly, and often incomplete, limiting its routine use.

Another approach to density separation is the use of denser liquids for separating MPs. The buoyancy of oil has been exploited, particularly in soil or sediment samples, and canola oil has been applied to separate MPs in liquid samples (Sheriff et al. 2024; Zhao et al. 2024). Furthermore, more concentrated solutions such as filtered lithium metatungstate (LMT, 1.62 g/mL) and sodium polytungstate (SPT) have also been used for density separation (Sheriff et al. 2024). The density of aqueous SPT solutions ranges from 1.0 to 3.1 g/cm^3^, depending on the concentration, an intermediate density between sediments and plastics. Their ease of recovery and non-toxic nature have contributed to the widespread use of SPT and LMT (Sheriff et al. 2024; Uguagliati et al. 2023).

Once digestion and separation are complete, samples are filtered through gold-plated polycarbonate or stainless-steel filters with pore sizes smaller than the target MP size, followed by air drying (Ribeiro-Claro et al. 2017; Schymanski et al*.* 2017).

Overall, sample preparation methods should be optimized for each matrix. For water and air samples, direct filtration using gold-coated polycarbonate or stainless-steel filters (~ 1.2 µm) should be standardized, while staining methods such as Nile Red should be considered supplementary rather than primary detection tools. For sediments, ZnCl₂-based density separation provides the most reproducible recovery and should be coupled with transparent reporting of solution parameters to enhance comparability across studies. For biological matrices, 10% KOH digestion at 60 °C for 24 h is recommended as the baseline protocol, while Fenton’s reagent serves as an effective alternative for more resistant tissues. Enzymatic digestion should be reserved for samples where maximal MP preservation is required.

Although these preparation techniques are effective for their respective matrices, inconsistencies among studies remain a significant limitation. Establishing harmonized quality assurance and quality control (QA/QC) procedures, including standardized filter materials, digestion reagents, and density separation protocols, would significantly improve reproducibility and comparability of Raman spectroscopy data on MPs across different environmental matrices.

## Raman-based techniques for MP detection and characterization

Raman spectroscopy is a powerful analytical technique used to identify and characterize MPs based on their molecular vibrations. In 1928, Indian physicist C.V. Raman discovered that a small fraction of light scattered by certain molecules exhibits a shift in wavelength compared to the incident light, a phenomenon now known as Raman scattering (Dabrowska et al. 2021; Unnimaya et al. 2023). This shift is influenced by the chemical structure of the scattering molecules. Raman spectroscopy operates by irradiating a sample with a monochromatic laser and measuring the scattered light at a specific angle. When no light absorption occurs, elastic (Rayleigh) scattering dominates. However, a small fraction of light undergoes inelastic scattering (Raman scattering), producing a spectrum with peaks corresponding to molecular bond vibrations (e.g., C–C, C = C, N–O, C = O, C-H), making the technique ideal for analyzing polymer chains.

Raman spectroscopy is possibly non-destructive and capable of analyzing solid, liquid, and biological samples without causing damage, making it especially valuable for MP characterization. Technological advances have improved sensitivity, enabled faster measurements, and enhanced detection of smaller particles. The development of handheld Raman spectrometers has facilitated in situ MP analysis without extensive sample preparation (Donato et al. 2024; Iri et al. 2021).

Raman spectroscopy can quantify MPs in the size range of approximately 1 µm down to the sub-micrometer scale; however, its performance is limited when analyzing samples with strong autofluorescence, which can obscure Raman signals.

### Fundamentals of Raman spectroscopy

It should be noted that Raman spectrometry could be destructive, as laser excitation induced photodegradation of phthalocyanine dyes encapsulated in microcapsules and coated particles (Bratashov et al. 2011). Studies on copper phthalocyanine (CuPc) paint layers further demonstrated that nanosecond pulsed irradiation during Raman analysis caused rapid local heating, leading to phase transitions and irreversible structural changes. In contrast, continuous-wave excitation was less damaging, indicating a higher potential for thermal degradation under pulsed excitation (Balakhnina et al. 2024; França de Sá et al. 2016).

In recent years, artificial intelligence (AI), particularly machine learning (ML), has become a valuable tool in analytical sciences, including Raman spectroscopy (Qi et al. 2023). ML efficiently handles complex, high-dimensional datasets and identifies patterns without extensive prior knowledge. Support vector machines (SVMs) are widely used to classify and regress nonlinearly separable MP data by maximizing class separation and minimizing errors (Qi et al. 2023; Sunil et al. 2024b). Principal component analysis and linear discriminant analysis (PCA LDA) simplify complex datasets and improve class distinction for more accurate classification. This approach first reduces dimensionality through PCA and then enhances separation using LDA (Qi et al. 2023; Sunil et al. 2024a). Artificial neural network (ANN) algorithms complement traditional methods such as LDA, linear regression, and spectral preprocessing by performing dimensionality reduction and data optimization (Qi et al. 2023). Collectively, these techniques enable automated classification of Raman spectra, improve analytical efficiency, and expand opportunities for research (Sunil et al. 2024b).

One of the latest advancements is the development of portable handheld Raman spectrometers for the analysis of MPs/NPs in the field (Chakraborty et al. 2022; Farale et al. 2025). These devices enable sample analysis without long-distance transportation or the need for a laboratory environment (Farale et al. 2025). However, handheld Raman spectrometers require further improvement in sensitivity, specificity, and operating capacity under various environmental conditions for effective in situ MPs/NPs analysis (Farale et al. 2025). Moreover, although portable devices are more affordable, their sensitivity and resolution remain inferior to benchtop instruments (Farale et al. 2025).

CARS, in particular, enables subcellular analysis of MPs without extensive sample processing, providing valuable insight into particle uptake and localization within biological tissues. In addition to its technical benefits, Raman spectroscopy is considered environmentally friendly. The method has low energy demands, requires no chemical solvents or reagents, and generates no hazardous waste, making it a sustainable choice for environmental analysis. However, the alignment and calibration of Raman instruments can be time-intensive, and potential gains in simplified sample preparation may be offset by the complexity of the instrumentation (Ribeiro-Claro et al. 2017).

### Core applications of Raman to analyze MPs

CARS, an advanced technique derived from Raman spectroscopy, addresses this issue by producing high-contrast, laser-like signals through a nonlinear optical process. CARS enables detailed 2D or 3D imaging of MPs, particularly useful in biological and environmental samples such as soil and marine ecosystems (Ribeiro-Claro et al. 2017; Rhee et al. 2024). For example, CARS has been used to distinguish PS particles from natural biological materials and to detect MPs in complex soil matrices. Polymers like PMMA, PA, PE, and PS have been successfully analyzed with CARS microspectroscopy. Despite its advantages, the CARS technique also has several limitations. It suffers from inherently slow imaging speeds due to detector and sample scanning constraints, which negatively impact its ability to capture the dynamic processes of MPs in organismal cells (Choi et al. 2022). Furthermore, the CARS technique has limited penetration depth in thick biological tissues, which further restricts its application (Choi et al. 2022). The complexity of ultrafast laser systems, their high cost, and the need for specialized and regular maintenance make the CARS technique difficult to use routinely (Choi et al. 2022; Ribeiro-Claro et al. 2017). In addition, the development of comprehensive CARS spectral libraries for identifying different MP species remains a significant need in this field (Rhee et al. 2024).

SERS is another sensitive technique that amplifies weak Raman signals, enabling detection of low concentrations of MPs and nanoplastics (NPs). Discovered in 1974, SERS leverages two mechanisms: the electromagnetic mechanism, based on localized surface plasmon resonance (LSPR), and the chemical mechanism, involving charge transfer interactions under laser excitation (Mika et al. 2022). Factors such as metal type (silver or gold), nanoparticle size, and shape influence SERS effectiveness. Recent studies have shown that SERS can detect plastics like PS and PET at nanometer scales using substrates such as gold nanoparticles and silver colloids (Mika et al. 2022; Luo et al. 2023; Shorny et al. 2023). Despite its potential, SERS is underutilized for MP/NP detection due to challenges in substrate reproducibility and signal variability. Future research should focus on improving substrate consistency and analytical sensitivity (Luo et al. 2023).

CRT is a novel approach enabling rapid imaging and quantification of MPs. CRT uses fast-programmable spectral filters and single-pixel detectors, achieving up to 100 × faster analysis than conventional Raman methods. With pixel dwell times as low as 250 µs and 1 µm spatial resolution, CRT provides both morphological and chemical information about MPs and is adaptable for underwater, field, and laboratory use (Grand et al. 2024). However, it requires expertise in binary filter design and specialized algorithms, and its effectiveness is limited when compounds are unknown or complex.

The continuous breakdown of MPs into smaller particles through environmental stress increases their potential impact on biological tissues. While a light or a polarization microscopic technique can detect micron-sized MPs, they lack the resolution for full identification. Micro-Raman spectroscopy offers rapid and detailed chemical and morphological characterization (Frere et al. 2016; Zhang T et al. 2023). Combined with optical microscopy, Raman methods can detect submicron particles and provide high spatial resolution. Bright background interference remains a limitation, especially in biological samples. Integrating Raman with scanning electron microscopy (SEM) has enabled the detection of MPs as small as 100 nm, and automation tools like particle recognition software have reduced analysis time (Sarau et al. 2020; Tsering et al. 2022).

Fluorescent labeling, unlike Raman area scans, increases the probability of detecting small MPs even at low concentrations or in clumped distributions (Krekelbergh et al. 2025; Le et al. 2025). This approach provides almost instantaneous analysis, high spatial and temporal resolution, and avoids interference from background MP contamination. However, it is limited to controlled experiments in which fluorescently labeled MPs are intentionally added, making it unsuitable for detecting naturally occurring environmental MPs. Despite this limitation (Krekelbergh et al. 2025), it remains a powerful technique for studying the dynamics of MPs infiltrating soil layers (Krekelbergh et al. 2025; Le et al. 2025).

Convolutional neural networks (CNNs) are being applied to improve Raman spectroscopy data analysis. Unlike traditional methods, CNNs automatically extract meaningful features from spectral data, handling background noise and enhancing signal interpretation. CNNs have demonstrated high accuracy in identifying MPs, such as PE, across different water matrices, providing a reliable alternative to manual data processing (Ren et al. 2023; Luo et al. 2024).

Raman spectroscopy has been successfully used to detect MPs in diverse environments, including terrestrial, marine, polar, and biological systems (Loughlin et al. 2021). Table 2 below summarizes the application of Raman spectroscopy for common MP types.

**Table 2:** Summary of Raman spectroscopy applications for common MPs

MP type	Detection methods	Size range	Applications	Ref
Polystyrene	Raman, SERS, CARS	< 100 nm to > 20 µm	Soil, biological tissues, water, air	Araujo et al. (2018); Osoro et al. (2024); Rhee et al. (2024)
Polyethylene	Raman, CARS, CRT, CNN-enhanced	~ 100 nm to > 20 µm	Marine water, soil, biological samples	Phan et al. (2022); Grand et al. (2024);
Polyamide	CARS, Raman	µm-scale	Sediment, biological tissues	Genchi et al. (2023); Rhee et al. (2024)
Polyester	Raman, SERS	Nanometer to µm-scale	Clothing fibers, indoor air, wastewater	Bottari et al. (2022); Li et al. (2025)
Polypropylene	Raman	µm-scale	Consumer products, surface water	Phan et al. (2022); Perraki et al. (2024); Jung et al. (2024)
PET	Raman, SERS	Nanometer to µm-scale	Bottled water, packaging, wastewater	Schymanski et al. (2017); Xing et al. (2025); Zhang J et al. (2023)
PMMA	CARS, Raman	Nanometer to µm-scale	Environmental samples, lab studies	Rhee et al. (2024)
PVC	Raman	µm-scale	Industrial waste, sediment	El Khatib et al. (2023); Seewoo et al. (2024)
Others	Raman, CARS, SERS	Variable	Environmental matrices and complex mixtures	Araujo et al. (2018); Ciornii et al. (2025)

Techniques other than Raman spectrometry are also employed for the analysis of MPs and NPs in various environmental samples. Although these techniques each have advantages and limitations, their combined application is often preferred to improve the precision and accuracy of MPs analysis results (Huang Z et al. 2022).

Mie theory, unlike light scattering techniques traditionally used for MP analysis, is based on the scattering of individual particles. It provides a theoretical framework that explains how particles in aquatic environments scatter light, allowing particle size, refractive index, and density to be determined from the angular distribution and intensity of scattered light (Wang et al. 2024). Raman spectroscopy and Mie scattering differ in their analytical applicability. Raman spectroscopy provides molecular fingerprints through vibrational spectra and can identify polymer species in environmental samples, whereas Mie scattering is sensitive to particle size and concentration and can detect particles as small as 1–10 µm but lacks polymer-specific identification capability (Genc et al. 2023, 2024).

FT-IR is another vibrational spectroscopic technique widely used for MPs analysis. Compared with Raman spectroscopy, FT-IR can typically analyze particles larger than 20 µm due to its spatial resolution of 10–20 µm, while Raman can detect particles as small as 500 nm (Huang Z et al. 2022). Both techniques exhibit high polymer specificity; however, Raman spectroscopy generally resolves narrower bands and is more sensitive to nonpolar functional groups, whereas FT-IR is more effective for polar groups (Huang Z et al. 2022). FT-IR requires completely dry MP samples to prevent water interference, while Raman spectroscopy can be applied directly to wet samples without dehydration (Araujo et al. 2018). For environmental matrices, FT-IR is less affected by autofluorescence interference from contaminants on the MP surface or additives in the plastic matrix but cannot effectively analyze black materials. In contrast, Raman spectroscopy can analyze dark-colored MPs but is more prone to fluorescence and photodegradation (Huang Z et al. 2022).

Hyperspectral imaging (HSI) is another technique used to detect MPs in environmental matrices, offering distinct advantages and limitations compared with Raman spectroscopy. Raman spectroscopy provides high spatial resolution, with detection limits as low as 1 µm, whereas HSI is less sensitive at the microscale, typically detecting MPs down to 50–300 µm in water and about 100 µm in soils. Sensitivity in HSI is strongly influenced by particle size, color, and the surrounding soil matrix (Ali et al. 2024). Raman analysis is more susceptible to fluorescence interference from contaminants and additives and generally requires labor intensive sample preparation steps, including density separation, digestion, and filtration, to remove organic matter and ensure reliable detection (Ali et al. 2024). In contrast, HSI requires minimal pretreatment, such as drying and sieving, and can often analyze MPs directly in soils, making it faster and easier to implement than Raman spectroscopy. However, environmental factors such as soil texture, moisture content, particle size, and polymer color can reduce HSI performance, leading to nonlinear spectral responses (Ali et al. 2024).

Near infrared (NIR) spectroscopy is another rapid, possibly non-destructive technique for polymer analysis. However, Raman spectroscopy generally provides more reliable results for polymer identification and can distinguish similar polymers, such as LDPE and HDPE. While Raman spectroscopy can detect MPs as small as 1 µm and even approach the submicron range (approximately 200 nm using advanced line scanning systems), NIR has lower spatial resolution and is less suitable for nanoscale particle analysis. Sample preparation for both Raman and NIR is minimal and possibly non-destructive, but Raman spectroscopy is more prone to fluorescence interference due to additives and surface contaminants on MPs (Huang Z et al. 2022).

Table 3 offers a concise comparative framework for evaluating Raman-based methods in MP detection, highlighting their analytical attributes across parameters such as detection limit, speed, sample compatibility, and key limitations.

**Table 3:** Major Raman-based analytical advances for MP detection

Technique	Analytical focus	Detection range/resolution	Advantages	Limitations	Ref
Conventional Raman	Polymer identification via vibrational fingerprint	~ 1 µm–5 mm	Non-destructive, high molecular specificity	Fluorescence interference, slower mapping	Ribeiro-Claro et al. (2017)
CARS	Non-linear Raman imaging	~ 0.3–1 µm	Label-free 2D/3D imaging, high contrast	Complex setup, limited penetration depth	Rhee et al. (2024); Choi et al. (2022)
SERS	Surface-enhanced signal via plasmonic substrate	< 1 µm (NP scale)	Very high sensitivity, trace detection	Substrate reproducibility issues	Mikac et al. (2023); Luo et al. (2023)
CRT	Fast spectral compression and imaging	~ 1 µm/250 µs pixel dwell	100 × faster mapping, chemical-morphological info	Requires advanced algorithms	Grand et al. (2024)
Raman–SEM	Combined morphology and composition	~ 100 nm	Sub-micron particle detection	Time-consuming, costly	Sarau et al. (2020); Tsering et al. (2022)
Fluorescent labeling	Tracking of tagged MPs	< 10 µm	High spatial–temporal resolution	Only for controlled experiments	Krekelbergh et al. (2025); Le et al. (2025)
AI-assisted Raman (CNN, ANN, PCA-LDA)	Automated spectral classification	Variable	Rapid, noise-resistant identification	Requires training datasets	Qi et al. (2023); Sunil et al. (2024b)
Portable/handheld Raman	Field and on-site detection	~ 5–20 µm	Immediate analysis, minimal prep	Lower sensitivity vs. benchtop	Chakraborty et al. (2022); Farale et al. (2025)

### Challenges of using Raman to analyze MPs

Fluorescence and photodegradation are among the most persistent challenges in Raman spectroscopy of MPs. Fluorescence often overwhelms the relatively weak Raman signal, particularly in environmentally aged or pigmented polymers (Hengstmann and Fischer 2019). Surface oxidation, adsorption of dissolved organic matter, and the presence of dyes or additives increase background fluorescence, masking the diagnostic vibrational bands required for accurate polymer identification (Hengstmann and Fischer 2019; Prata et al. 2021). Prolonged laser exposure can also induce photodegradation, leading to oxidation or polymer chain scission that alters the spectra (Gachayzade et al. 2025). These effects typically manifest as band broadening, peak shifts, or reduced intensities, thereby complicating spectral matching and classification.

Weathering-related spectral changes are particularly evident in reference polymers such as PE, PP, and PET. In PE, environmental oxidation introduces carbonyl stretching bands near 1715 cm⁻^1^, while PP shows new hydroxyl-related bands around 3400 cm⁻^1^ after UV exposure. PET typically exhibits decreased intensity in its characteristic C = O stretching band near 1725 cm⁻^1^ following photodegradation (Hengstmann and Fischer 2019). These spectral alterations highlight the limitations of conventional reference libraries, which are primarily built from pristine materials and may yield poor match scores or misclassifications when analyzing weathered MPs (Shruti and Kutralam-Muniasamy 2023).

In addition to photochemical effects, analytical biases introduced during sample processing can influence Raman outcomes. Procedural blanks, often exposed to airborne or laboratory contamination, can bias results if not properly monitored (Shruti and Kutralam-Muniasamy 2023). Despite their importance, blank contamination and bias are frequently underreported. Establishing standardized QA/QC protocols, including blank correction, calibration of reference spectra, and validation of spectral matches, is critical to ensure reproducible Raman data across laboratories (Shruti and Kutralam-Muniasamy 2023).

Fluorescent dyes such as Nile Red, commonly used for MP prescreening, improve throughput by facilitating the pre-identification of polymeric particles. However, they also introduce background fluorescence that can interfere with Raman analysis if not adequately corrected (Hengstmann and Fischer 2019; Prata et al. 2021). Consequently, when Nile Red is used, spectra must be interpreted cautiously, or dye-free verification steps should be incorporated.

Despite these advances, several analytical and methodological challenges continue to limit the full standardization of Raman spectroscopy for MP analysis. Fluorescence interference remains the most persistent obstacle; fluorescent backgrounds generated by pigments, additives, or humic substances often obscure Raman signals in complex matrices (Araujo et al. 2018; Ribeiro-Claro et al. 2017). Near-infrared excitation and controlled photobleaching can reduce this interference but may introduce thermal degradation or spectral distortion when poorly optimized.

Another major limitation is spectral variability among polymers. Environmental aging, UV-induced oxidation, and biofouling alter characteristic vibrational bands, such as carbonyl and hydroxyl groups appearing in weathered PE, PP, and PET, which complicates spectral classification (Shruti & Kutralam-Muniasamy 2023). Expanding spectral libraries to include weathered and pigmented MPs is therefore essential.

Particle-size constraints also affect detection accuracy. Raman spectroscopy identifies ≈ 80% of MPs larger than 100 μm but fewer than 65% of particles below 50 μm due to weak scattering and reduced signal-to-noise ratios (Farale et al. 2025). Interlaboratory reproducibility remains problematic, with variability exceeding 30% across laboratories (Ciornii et al. 2025). Detection limits are also matrix-dependent, 1–10 μm for water samples but 50–200 μm for sediments, hindering cross-study comparison (Lao & Wong 2023).

Data comparability is weakened by inconsistent preprocessing and spectral-matching approaches. Variations in baseline correction, normalization, and algorithm selection can yield divergent polymer identifications (Ciornii et al. 2025). Addressing these methodological inconsistencies is imperative for reproducible and regulatory-grade Raman analyses.

### Mitigation strategies and complementary approaches

Several strategies have been proposed to mitigate fluorescence and photodegradation in Raman spectroscopy of MPs. Instrumental optimization—using lower laser power, shorter integration times, and near-infrared excitation wavelengths—can minimize photothermal damage to polymer surfaces (Gachayzade et al. 2025). Pretreatment procedures such as oxidative digestion and density separation help remove organic matter that contributes to autofluorescence, while Raman spectral preprocessing (e.g., baseline subtraction, smoothing, and normalization) enhances signal interpretation. Combining Raman spectroscopy with complementary techniques such as FTIR or thermal analysis further improves the identification of weathered MPs by providing orthogonal data on polymer composition and degradation states. Together, these advances contribute to a more robust interpretation of problematic spectra and improved discrimination between pristine and aged MPs.

## Environmental occurrences and Raman applications for MP profiling

With the widespread presence of MPs in the environment, plastic waste presents a persistent and long-term challenge to ecosystems worldwide. While microscopic techniques provide some insight, they are inadequate for thoroughly investigating the sources and compositions of MPs, thereby necessitating the use of advanced spectroscopic tools. This review focuses on the broad applications of Raman spectroscopy in characterizing MPs across various ecosystems. Table 4 summarizes environmental occurrences and Raman-based detection of MPs.

**Table 4:** Environmental occurrences and Raman-based detection of MPs

Environment/matrix	Common MP types detected	Detection technique(s)	Key findings/notes	Ref
Ocean surface/marine	PES, PET, PA, PE, PP, PS	Raman, SRS	MPs found in surface waters, sediment, beaches, deep sea	Aramendia et al. (2024); Caldwell et al. (2023); Faull et al. (2024); Karami et al. (2017); Kazour et al. (2019); Kumar et al. (2021); Saad and Alamin (2024); Sambandam et al. (2022); Schroder et al. (2021)
Freshwater systems	PE, PP, PS	Raman	Sources include WWTPs, residential runoff	Lavoy and Crossman (2021); Montono-Martinez et al. (2021); Saad and Alamin (2024); Saad et al. (2022); Viitala et al. (2022)
Aquatic organisms	PE, PS, PP, PES	Raman	Ingestion observed from fish to whales; neurotoxic effects noted	Barboza et al. (2023); Caldwell et al. (2023); Manolaki et al. (2023); Saad and Alamin (2024); Thiele et al. (2021)
Seafood (for human use)	PE, PS, PP	Micro-Raman	MPs detected in mussels and shellfish	Kumar et al. (2021); Manolaki et al. (2023)
Human exposure (dietary)	PE, PA, PP, PET, PVC, PVOH, PS, PES, PC	Raman, SERS	Found in breast milk, baby food, kidneys, liver, and GI tract	Ho et al. (2022); Kadac-Czapska et al. (2023); Liu M et al. (2023); Massardo et al. (2024); Ragusa et al. (2022); Zhang J et al. (2023)
Dairy and beverages	PE, PP, PES, PTFE	Raman	MPs from equipment and packaging found in milk and bottled water	Da Costa Filho et al. (2021); Schymanski et al. (2017); Winkler et al. (2022)
Salt/condiments	PET, PP, LDPE, Nylon 6	Raman	Open-pan salts contain up to 7216 MPs/kg	Mazumder et al. (2023)
Tea bags/hot drinks	PE, PET, PP, Nylon 6	Raman	MPs released during use regardless of temperature	Mei et al. (2022)
Indoor air/PM10	PE, PS, PET, ABS	Raman, SERS	MPs prevalent in indoor environments and urban aerosols	Gaston et al. (2020); Yoo et al. (2023)
Soil/agriculture	LDPE, PE, PP	Raman	MPs from plastic mulch and greenhouse covers impact soil and plant uptake	Sorasan et al. (2024); Tympa et al. (2021)
Atmospheric aerosols	PS, PMMA	SERS	Detected using Clarite and TCA substrates	Kundu et al. (2021); Xu et al. (2020); Zhang T et al. (2023)
Groundwater	Not specified	Raman	MPs prevalent in shallow aquifers	Perraki et al. (2024)
Polar regions	PES, PP	Raman	MPs found in Antarctic fish, signaling global spread	Bottari et al. (2022)

### Aquatic environments

Due to the higher mobility and ease of dispersion of MPs in water compared to soil, aquatic environments have received particular attention. The transparency of water makes sample preparation procedures easier than those for soil, sediment, and organism samples, which have density ranges similar to MPs and can interfere with spectroscopic techniques (Rani et al. 2023). MPs have been detected in ocean surfaces, water columns, sediments, beaches, rivers, and even in deep-sea regions (Aramendia et al. 2024; Liu et al. 2022; Ripken et al. 2021; Sambandam et al. 2022; Schroder et al. 2021). Stimulated Raman microspectroscopy (SRS) has been employed to identify chemical compositions and abundances of microfibers, especially PES, PET, and PA, in marine environments, revealing their anthropogenic and domestic origins (Caldwell et al. 2023; Faull et al. 2024; Karami et al. 2017; Kazour et al. 2019; Kumar et al. 2021; Sadd and Alamin 2023; Sambandam et al. 2022). In freshwater systems, major sources of MPs include wastewater treatment plants and human activities in residential areas (Lavoy and Crossman 2021; Sadd and Alamin 2023; Sadd et al. 2022; Viitala et al. 2022).

The ingestion of MPs by aquatic organisms, from small fish to whales, demonstrates their ecological threat (Barboza et al. 2023; Caldwell et al. 2023; Manolaki et al. 2023; Montoto-Martinez et al. 2021; Saad and Alamin 2024; Thiele et al. 2021). Behavioral changes, including altered swimming and feeding patterns due to neurotoxicity, have been reported in fish exposed to MPs (Barboza et al. 2023). These effects raise concerns about potential neurotoxicity in humans. Floating plastics such as PE, PS, and PP (less dense than seawater) are easily ingested by marine organisms (Caldwell 2023; Kundu et al. 2021). MPs have also been found in commercial seafood, including mussels and shellfish consumed by humans (Kuma et al. 2020; Manolaki et al. 2023). In one study, fragments and fibers were discovered in the stomachs of tiger sharks in the Atlantic, indicating top predator exposure to plastic waste (Munno et al. 2024).

Drinking water treatment plants commonly contain PP, PET, and PE fibers (Adib et al. 2021; Chakraborty et al. 2022). Micro-Raman spectroscopy has been used to characterize 25 nm PS nanospheres as well as MPs larger than 5 µm, such as PS and PE (Bereczki et al. 2023; Frere et al. 2016; Trainic et al. 2020). In mangrove forests, MPs reflect pollution from fishing and tourism (Valsan et al. 2024). PES and PP MPs have been found in Antarctic fish species, underscoring the global scale of plastic pollution (Bottari et al. 2022).

### Terrestrial environments

Various sources, including sewage sludge, agricultural mulch, compost, atmospheric deposition, road dust, and degraded plastics, increasingly contribute to MP contamination in soils (Chen et al. 2024; Kang et al. 2025). The accumulation of MPs in soil alters physical properties such as porosity, pH, and water retention, thereby disrupting the soil carbon cycle and microbial communities (Chen et al. 2024; Kang et al. 2025). MPs also inhibit seed germination and root elongation and reduce photosynthetic activity by disturbing the chlorophyll a/b ratio (Kang et al. 2025; Huang D et al. 2022). The cumulative effects of MPs degrade soil health, diminish agricultural productivity, and pose ecological risks (Kang et al. 2025; Huang D et al. 2022). Furthermore, MP interactions with other contaminants can alter microbial communities and enzyme activities and increase plant stress, thereby influencing the soil carbon pool and terrestrial plant performance (Chen et al. 2024; Kang et al. 2025).

Agricultural plastics, such as greenhouse covers made from LDPE, release MPs under sunlight, contaminating soil and water and potentially affecting crops (Sorasan et al. 2024). MPs have been found in lichen near landfills and in plant tissues, such as radishes exposed to ABS MPs (Loppi et al. 2021; Tympa et al. 2021). Groundwater, particularly shallow aquifers, has been contaminated with MPs (Perraki et al. 2024).

### Atmospheric environments

MPs are also prevalent in the air, especially in indoor environments, where they originate from clothing and domestic sources (Gaston et al. 2020). MPs such as PS, PE, PET, and ABS have been detected in PM10 aerosol samples from urban areas (Yoo et al. 2023). Although there is limited information on the behavior and fate of MPs in the atmosphere, available evidence suggests that they can be transported, accumulated, and present in both indoor and outdoor environments (Kernchen et al. 2024; Rahman et al. 2021). The main sources of atmospheric MPs are road dust, emissions from residential buildings, and industrial activities (Kernchen et al. 2024; Munno et al. 2020; Worek et al. 2022). Road dust is an important reservoir of tire-derived MPs due to direct emissions from braking and abrasion, with TWPs potentially accounting for over 50% of primary MPs in some regions (Worek et al. 2022). In atmospheric MP pollution studies, carbon black, detected in outdoor air samples, can mask the analysis of MPs, as it overwhelms key spectral regions and shares structural similarities with soot (Rahman et al. 2021; Kernchen et al. 2024).

Carbon black, used as a dye and reinforcer in plastics and rubber, primarily originates from tire wear particles (TWPs) in traffic emissions (> 90% of environmental particles), where it mixes with soot and particulates; road dust shows higher MP concentrations than adjacent soils due to direct deposition (Kernchen et al. 2024; Rahman et al. 2021; Worek et al. 2022). Raman spectroscopy excels for airborne MP analysis by distinguishing TWPs from soot via targeted spectral regions and preprocessing (Rahman et al. 2021; Zhang et al. 2024). Carbon black’s signature features broad disorder and graphitic bands that often dominate the fingerprint region, causing signal distortion (Rahman et al. 2021; Worek et al. 2022). Soot overlaps in these bands but lacks polymer-specific vibrations (e.g., rubber stretches); differentiation involves masking the interfering region and targeting less affected C–H stretches, achieving > 70% hit quality for SBR in TWPs (Rahman et al. 2021; Munno et al. 2020). TWP-specific peaks, such as CH₂ stretches, C–H ring stretches, and ring breathing modes, aid distinction from soot (Worek et al. 2022; Zhang et al. 2024). Integrating these fingerprints with libraries like SLoPP-E improves accuracy for sub-10 µm inhalable fractions, reducing underestimation (e.g., vs. FTIR) and enabling standardized QA/QC (Munno et al. 2020; Rahman et al. 2021; Kernchen et al. 2024). SERS with Clarite substrates has also detected atmospheric PS and PMMA aerosols (Kundu et al. 2021; Xu et al. 2020).

### Biological and human exposure

In humans, ingestion is the primary route of MP exposure. MPs have been detected in everyday items such as salt, sugar, bottled water, milk, tea bags, and disposable utensils (Gambino et al. 2023; Mei et al. 2022; Schymanski et al. 2017; Thiele et al. 2023; Winkler et al. 2022). Raman analysis of plastic-coated paper cups confirmed that MPs such as PE and PA migrate into hot drinks (Zhang T et al. 2023). MPs ranging from 2 to 12 µm, including PE, PVC, PP, PS, PES, polycarbonate (PC), and polyvinyl alcohol (PVOH), have been found in breast milk, and PA, PE, PP, and PET in baby food (Kadac-Czapska et al. 2023; Ragusa et al. 2022). A Hong Kong cohort study found MPs in the human gastrointestinal tract, particularly among individuals with high seafood consumption (Ho et al. 2022). MPs like PE and PS have also been detected in human kidneys and urine, and PP MPs were observed in mouse liver tissues, correlating with changes in lipid metabolism (Liu M et al. 2023; Massardo et al. 2024).

In dairy, MPs such as PE (from milking machines) and others like PP, PES, and PTFE (from storage containers) were found in raw cow milk (Da Costa Filho et al. 2021). Packaged dairy products and bottled water also show plastic contamination (Da Costa Filho et al. 2021; Schymanski et al. 2017; Winkler et al. 2022). Interestingly, more MPs were found in water from glass bottles than PET bottles, likely due to PE leaching from caps (Gambino et al. 2023). Salt contamination is another concern; open-pan salts have shown MP levels ranging from 2700 to 7216.1 MPs/kg, attributed to environmental exposure during production (Mazumder et al. 2023). Raman spectroscopy has detected PET, PP, low-density polyethylene (LDPE), and Nylon 6 in salt production and packaging materials, as well as in tea filter bags, which released MPs into water regardless of temperature or soaking time (Mazumder et al. 2023; Mei et al. 2022).

SERS can detect PS particles as small as 50 nm and has also been used to analyze PET and PP in baby bottles, bottled water, and tap water (Luo et al. 2023; Zhang J et al. 2023). Specialized substrates, such as triangular cavity arrays (TCA), enable detection of PET NPs as small as 200 nm in drinking water (Zhang J et al. 2023). This technique can even analyze MPs without pretreatment by using spongy substrates with Au nanoparticles (Yin et al. 2021). The SERS technique has proven especially effective in detecting MPs and NPs. Using Au nanoparticle substrates, 350 nm PS spheres were detected with a detection limit of 6.25 µg/mL (Mikac et al. 2023).

## Raman spectral profiles of common MP polymers

### Polystyrene

Table 5 presents data from the analysis of PS using Raman spectroscopy, based on samples collected from various sources. PS is a lightweight, low-cost plastic commonly used in insulation materials, packaging, and kitchen utensils. However, its widespread use has led to significant accumulation in landfills and the environment. As PS breaks down into smaller fragments, it poses risks to both terrestrial and marine life—highlighting the importance of Raman spectroscopy in MP identification and characterization. Figure S1 displays the Raman spectrum of PS (Rytelewska and Dabrowska 2022). Key peaks used to identify PS include the 621 cm⁻^1^ bending vibration of carbon atoms in the benzene ring, the 797 cm⁻^1^ symmetric ring-stretching mode, and the 1000 cm⁻^1^ aromatic C–C ring stretching (Sunil et al. 2024a; Xue et al. 2022).

**Table 5:** Raman spectral profile of PS MPs

Source	Size (µm)	Raman fingerprint (cmˉ^1^)	Band assignment	Country/year	Ref
Fresh water and WWTP	> 1	1000 2800–3000	-CH bending vibration-aromatic ring -CH vibration	Poland/2022	Rytelewska and Dabowska (2022)
Marine sediment	> 0.45	1002, 1610, 3059	-	China/2022	Liu et al. (2022)
Plastic consumables	13–250	620, 997, 1028, 1600	C–C-C (radial ring deformation) C–C (str. radial) C-H in plane bending C–C str	India/2023	Unnimaya et al. (2023)
PS nanospheres	From 500 to 25 nm	1002, 1031, 1603, 3057	Aromatic ring peak and C-H Stretch peaks	Brazil/2023	Bereczki et al. (2023)
MP particles	1 Polybead micron microspheres	1000, 3050	Aromatic ring peak and C-H Stretch peaks	Germany/2020	Sarau et al. (2020)
Aerosol samples	> 10	1000, 1600	-	USA/2020	Trainic et al. (2020)
Nanoplastic from Sigma Aldrich	800 nm, 300 nm, and 100 nm diameter spherical	1003	C–C stretch	Austria/2023	Shorny et al. (2023)
Seawater	1–5 mm fiber, fragments, film	620, 997, 1030, 1600, 3050	-	Greece/2023	Miserli et al. (2023)
Beach sediment	Particles and fibers > 500 μm	1000, 1600	-	Germany/2016	Kappler et al. (2016)
Human	30–1800 fragments	1000, 1040, 1610	-	Hong Kong/2022	Ho et al. (2022)
Human breastmilk	~ 2 µm Irregular fragments	-	-	Italy/2022	Ragusa et al. (2022)
Mediterranean Sea	1.2–3 filament, fragments, film	-	-	Poland/2023	Dabrowska et al. (2023)
Cow milk	≥ 5 µm Beads	1000, 1030, 1610	-	Switzerland/2021	Da Costa Filho et al. (2021)
Mussels	0.1–1 (diameter)	1000	-	Italy/2021	Valsesia et al. (2021)
Water	< 5 mm	622, 1002	Ring vibrations	India/2022	Chakraborty et al. (2022)
MP sample	< 10	1000, 3050	-	USA/2020	Primpke et al. (2020)
Wastewater sludge	Particle Fibers	-	-	Finland/2021	Asamoah et al. (2021)
MP samples	-	610, 1000	-	Belgium/2024	Plazas et al. (2024)
Beach sediment	0.2–5 mm Fragments	-	-	Germany/2021	Schroder et al. (2021)
Marine sediment	45–1.000 Spheres, fragment	-	-	USA/2022	Cashman et al. (2022)
Water and sediment	2–5	1003, 1033	C–C, C-H aromatic ring	USA/2022	Kundu et al. (2021)
Aquatic system	< 5 mm	1001 1602 3054	Aromatic ring Skeletal str C-H str. Benzene ring	Italy/2021	Nava et al. (2021)
Drink water plants	1–50	1000, 1600, 2850	-	Germany/2023	Maurizi et al. (2023)
MP samples	> 12	1465	-	Netherland/2018	Zada et al. (2018)
Air sample	20–850 Fiber-fragment	1003, 1030	-	USA/2020	Gaston et al. (2020)
Stock solution	50 nm	1001 1032	C–C ring mode C-H	Norway/2023	Luo et al. (2023)
Polystyrene (PS) nanospheres	50 nm	1003 1033	C–C and C-H ring mode	Norway/2023	Zhang T et al. (2023)
PM10 aerosols	> 10	1000, 1610, 2910, 3050	Aromatic ring peak and C-H Stretch peaks	S. Korea/2023	Yoo et al. (2023)
Drinking water DWTP	1–100 Fiber, fragment	-	-	Iran/2021	Adib et al. (2021)
MP samples	39–155	1002	-	China/2021	Yin et al. (2021)
Groundwater	> 20 Irregular particles	1002 1604 3060	C–C symmetric stretch C–C stretch C-H stretch on the benzene ring	Greece/2024	Perraki et al. (2024)
River, freshwater	1–5 mm Fragment, fiber	997, 1601, 2451, 2902, 3051	-	Romania/2024	Gheorghe et al. (2024)
Marine environment	> 12 PS beads	1000	-	France/2024	Grand et al. (2024)
WWTP	< 100 Fragments	620, 1000, 1030, 1600	-	Canada/2021	Lavoy and Crossman (2021)
MP samples	-	624 1004 1033 1605	C–C str C–C benzene ring vibr C-H vibr. Benzene ring CH str	China/2023	Ren et al. (2023)
Human kidneys and urine	3–13 fragment	620, 997	-	Italy/2024	Massardo et al. (2024)
Disposable fork	20–300	1000 3060	Aromatic C-H bending C-H str	Poland/2021	Dabrowska (2021)
Plastics samples	80–250 spherical	-	-	Finland/2024	Hrovat et al. (2024)
Mussel tissues	1–10 particles	1001, 1032, 1602	-	Spain/2024	Aramendia et al. (2024)
Marine environment	170–410 fibers	1000 3050	-	S. Arabia/2023	Genchi et al. (2023)
WWTP	> 20 fragment	-	-	Finland/2022	Tsering et al. (2022)
Ocean surface water	1.38–4.18	1000 1030 1610	C–C symmetric stretch Ring stretch C-H stretch on the benzene ring	Japan/2020	Ripken et al. (2021)
Water	10 beads	1035 3060	C-H stretch	Portugal/2018	Araujo et al. (2018)
IPWWTP	> 10	-	-	Germany/2023	Barkmann-Metaj et al. (2023)
Ocean	< 50 fragment	-	-	USA/2024	Faull et al. (2024)
Bivalve tissue	> 50 fragment	1000	-	UK/2019	Thiele et al. (2019)
Sea	0.335–5 mm Fragment, film	1000	-	France/2016	Frere et al. (2016)
Water	8.47	1000, 3000	-	Turkey/2021	Iri et al. (2021)
Mussel samples	3–60 Fibers, fragment	1000, 1600	-	Germany/2020	Kumar et al. (2021)
PS beads	1–2–5 spheres	1003 1033	C–C aromatic ring C-H aromatic	China/2020	Xu et al. (2020)
MP samples	< 5 mm	621 1001 1602	Bending vibration benzene Characteristic peak Stretching vibration C = C bonds	China/2022	Xue et al. (2022)
Marine and estuarine sediments	100–300 Film, fragment, sphere	-	-	USA/2023	El Khatib et al. (2023)
MP sample	350–1000 nm spheres	1005 SERS peak 1034 1605	Ring breathing vibration C-H plane deformation Ring skeletal str	Hungarian/2022	Mikac et al. (2023)
Marine environments	20–500 Fragments, fibers	-	-	France/2019	Kazour et al. (2019)
Paddy soil samples	9.5–11.5 Spherical beads	3050	-	S. Korea/2023	Rhee et al. (2024)
MPs + natural organic matter	10 sphere	617, 998, 1028, 1602, 3056	-	S. Korea/2025	Zou et al. (2025)
MP beads	4.21–101	2855, 2907, 3058	-	Germany/2023	Glockler et al. (2023)
Lake samples	20–80 microspheres	-		China/2023	Ma et al. (2023)
Marine environment	< 1 mm Fibers, fragments	1002, 1768, 3057	-	India/2022	Sambandam et al. (2022)
Seafood	< 1.6	-	-	France/2016	Dehaunt et al. (2016)
River/ocean shore sediments	0.063–5 mm	-	-	Italy/2023	Cofano et al. (2023)
MP samples	300–1000	1004, 1190, 1605, 2849, 2884, 3036	-	China/2024	Liu et al. (2024)

### Polyester

Table 6 presents data on PES analysis using Raman spectroscopy, based on samples collected from various sources. PES refers to a broad class of polymers containing the ester functional group (Feldman 2008). PES polymer fibers are widely used in clothing, upholstery, and other textile fabrics, as well as in industrial applications such as resins, films, and coatings (Feldman 2008). During laundering, PES fabrics release microfibers into wastewater systems, which are often ineffective at fully retaining those (Bottari et al. 2022). These textile microfibers, classified as MPs, can easily enter rivers and eventually reach seas and oceans (Bottari et al. 2022).

**Table 6:** Raman spectral profile of PES MPs

Source	Size (µm)	Raman fingerprint (cmˉ^1^)	Band assignment	Country/year	Ref
Human breastmilk	~ 5 µm fragment	1000, 1300, 1610	-	Italy/2022	Ragusa et al. (2022)
Fish	0.4–4.2 mm fibers	-	-	Italy/2022	Bottari et al. (2022)
Lichens	550–796 microfibers	632, 857, 1284, 1613, 1726	-	Italy/2021	Loppi et al. (2021)
Water	< 5 mm	1750	C = O str	India/2022	Chakraborty et al. (2022)
Marine sediment	45–1000 sphere, fragment, fiber	-	-	USA/2022	Cashman et al. (2022)
Sea sediment	0.2–5 mm Film, fiber	850, 1290, 1610, 1730	-	Russia/2020	Esiukova et al. (2020)
Tiger sharks GI tracts	45–355 fiber	860, 1100, 1300, 1620, 1740	-	USA/2023	Muno et al. (2023)
Lake water and sediment	> 11 fibers	-	-	Finland/2022	Viitala et al. (2022)
Surface sea water	10–20 fiber	1241, 1727, 2954, 3075	-	S. Arabia/2020	Laptenok et al. (2020)
Sea salt	30–150 fibers	850, 1280, 1610, 1750	-	UK/2023	Thiele et al. (2023)
Marine environment	170–410 fibers	1250, 1600, 1750	-	S. Arabia/2023	Genchi et al. (2023)
Glass bottles	5–50	-	-	Germany/2017	Schymanski et al. (2017)
Lagooning sludge	< 500 Fibers, fragments	-	-	Morocco/2020	El Hayany et al. (2020)
River-fresh water	0.5–5 mm Fiber, fragment	850, 1100, 1290, 1600	-	S. Africa/2022	Saad et al. (2022)
Marine and estuarine sediments	100–300 fibers, fragment	-	-	USA/2023	El Khatib et al. (2023)
WWTP	25–250 fibers	-	-	Czech Republic/2023	Spacilova et al. (2023)
River/ocean shore sediments	0.063–5 mm	-	-	Italy/2023	Cofano et al. (2023)

This group of MPs poses a serious environmental threat due to their ability to penetrate the tissues of living organisms (Bottari et al. 2022). Figure S2 displays the Raman spectrum of PES (Thiele et al. 2023), which exhibits several characteristic peaks corresponding to specific molecular vibrations. The peak at 701 cm⁻^1^ is attributed to C–C ring stretching, while the 857 cm⁻^1^ band corresponds to C–O–C bending. The 1093 cm⁻^1^ and 1284 cm⁻^1^ peaks are associated with C = O stretching and COO stretching, respectively. A prominent band at 1461 cm⁻^1^ represents CH₂ bending, and a strong-intensity peak at 1613 cm⁻^1^ is due to aromatic C = C stretching. Additionally, the spectrum shows a distinct C = O stretching band at 1726 cm⁻^1^ (Loppi et al. 2021).

### Polyethylene terephthalate

Table 7 summarizes Raman-spectroscopic data for PET MPs obtained from diverse environmental and industrial sources. PET is a durable PES used in everything from beverage bottles and food containers to textile fibers, medical supplies, electronic components, and automotive parts (Go et al. 2017). Its longevity means discarded items persist in the environment for extended periods (Gambino et al. 2023); although PET is technically recyclable, uncontrolled disposal has led to substantial MP pollution, where PET particles are typically found as fibers and fragments. Figure S3 presents a representative Raman spectrum of PET (Manolaki et al. 2023). Characteristic bands reflect vibrations of the terephthalate ring and ethylene–glycol moieties, including C–C–C in-plane bending at 628 cm⁻^1^, out-of-plane bending at 701 cm⁻^1^, and C–H out-of-plane bending at 790 cm⁻^1^. Additional peaks appear at 856 cm⁻^1^ (C–C breathing), 1114 cm⁻^1^ (C–H in-plane bending), 1175 cm⁻^1^ (combined C–H in-plane bending and C–C stretching), 1289 cm⁻^1^ (C(O)–O stretching), and 1316 cm⁻^1^ (C–H in-plane bending). Bands at 1411 cm⁻^1^ correspond to CCH and OCH bending, while aromatic C = C stretching is observed at 1572 cm⁻^1^ and phenyl C–C ring stretching at 1613 cm⁻^1^. The strong C = O stretching band appears at 1727 cm⁻^1^, followed by CH₂ stretching vibrations at ≈ 2800 and 3000 cm⁻^1^ and the phenyl C–H stretch at ≈ 3090 cm⁻^1^ (Kadac-Czapska et al*.* 2023; Sunil et al. 2024a).

**Table 7:** Raman spectral profile of PET MPs

Source	Size (µm)	Raman fingerprint (cmˉ^1^)	Band assignment	Country/year	Ref
Marine sediment	> 0.45	1619, 2972	-	China/2022	Liu et al. (2022)
Plastic consumables	10 µm–1 mm	633 855 1609 1725	C–C-C in-plane bend C–C vibration C = C str C = O str	India/2023	Unnimaya et al. (2023)
Sediment	< 400	Spectral range of 1580–1640	-	Germany/2016	Kappler et al. (2016)
Mussels and pearl oysters	0.8–1500 microfiber	1188, 1285, 1613, 1726	-	Greece/2023	Manolaki et al. (2023)
Human	30–1800 fibers	860, 1180, 1290, 1620, 1730	-	Hong Kong/2022	Ho et al. (2022)
Lichens	45–796 Fibers, fragments	632, 702, 858, 1616, 1728	-	Italy/2021	Loppi et al. (2021)
Dried fish	1–1000 Fragments, films	-	-	Malaysia/2017	Karami et al. (2017)
Fish meal	< 150 microfibers	-	-	UK/2021	Thiele et al. (2021)
Deep ocean	> 100 < 5	-	-	USA/2019	Choy et al. (2019)
Water	< 5 mm	1200	-	India/2022	Chakraborty et al. (2022)
MP sample	< 10	1610, 1720	-	USA/2020	Primpke et al. (2020)
Wastewater sludge	Particle fibers	-	-	Finland/2021	Asamoah et al. (2021)
MP samples	-	850, 1300, 1620, 1730	-	Belgium/2024	Plazas et al. (2024)
Aquatic system	< 5 mm	857 1615 1730	C-(O)-O str Ring mode C = O str	Italy/2021	Nava et al. (2021)
Drinking water plants	1–50	1610, 1730	-	Germany/2023	Maurizi et al. (2023)
Sea sediment samples	0.2–5 mm Fragment, film, fiber	625, 860, 1290, 1610, 1730	-	Russia/2020	Esiukova et al. (2020)
MP sample	> 12	1616	-	Netherlands/2018	Zada et al. (2018)
Salt	> 1.55	1295, 1460, 1731	-	Bangladesh/2023	Mazumder et al. (2023)
Tea filter bags	> 1 Fiber, fragments	860 1295 1617 1729	C(O)-O str CH₂ twisting C–C ring str C = O str	China/2022	Mei et al. (2022)
Baby bottle washing water	100 nm	1435–1460 2780–2980	-CH₂ bending -CH₂-CH₃ str. Vibr	Norway/2023	Luo et al. (2023)
Bottled drinking water	50–110 nm	1620 1760	C − O stretching C = O stretching vibration	Norway/2023	Zhang J et al. (2023)
Jellyfish	< 300	1620 1730	Aromatic ring viber C = O str	Switzerland/2023	Caldwell et al. (2023)
PM10 aerosols	1–10	1300, 1625, 1740, 3090	-	S. Korea/2023	Yoo et al. (2023)
DWTP	1–100 Fiber, fragments	-	-	Iran/2021	Adib et al. (2021)
MP samples	39–155	861, 1600	-	China/2021	Yin et al. (2021)
MP samples in water	> 20 powder	860, 1288, 1615, 1728, 2965, 3082	-	Germany/2024	Jacob et al. (2024)
Groundwater	> 20 Irregular particles	1612–1617 1726–1730 2883 3081	C–C ring of phenyl C = O str. vibration CH₂ str C-H of phenyl	Greece/2024	Perraki et al. (2024)
Marine environment	> 12 fragments	-	-	France/2024	Grand et al. (2024)
Tiger sharks GI tracts	45–355 fibers	-	-	USA/2023	Munno et al. (2024)
Disposable items	20–300	1300, 1720	-	Poland/2021	Dabrowska (2021)
Plastic samples	80–250	-	-	Finland/2024	Hrovat et al. (2024)
WWTP	> 20 fiber	-	-	Finland/2022	Tsering et al. (2022)
Water bottles	200 nm	-	-	Italy/2022	Winkler et al. (2022)
Water in different types of bottles	5–50	-	-	Germany/2017	Schymansky et al. (2017)
IPWWTP	> 10	-	-	Germany/2021	Asamoah et al. (2021)
Water bottles	15–180	859 1615 1728 3081	C–C, C-O str C = C str C = O str C-H str	China/2022	Jin et al. (2022)
Ocean	< 50	-	-	USA/2024	Faull et al. (2024)
Bivalve tissue	> 50 fragments	1720	-	UK/2019	Thiele et al. (2019)
Seawater and freshwater lakes	0.01–5 mm Fibers, fragments	630, 860, 1610, 1720	-	Poland/2023	Kaliszewicz et al. (2023)
Water bottled	10–100 fragments	-	-	Italy/2023	Gambino et al. (2023)
MP samples	Fibers, fragment	-	-	UK/2023	Kukkola et al. (2023)
Infant formulas	126–4380 Fibers, fragment	1152, 1259 1572 1720	C-O str C = C str. ring C = O str. ester	Poland/2023	Kadac-Czapska et al. (2023)
Mussel samples	3–60 fibers	1100, 1300, 1610	-	Germany/2020	Kumar et al. (2021)
Beach and sea water	15–900 fibers	628 856 1613 1727	C–C-C bond Breathing of C–C Phenyl group Str. C = O	India/2024	Sunil et al. (2024a)
Atmospheric samples extracted	< 1 particle	633, 858 1300 1620, 1760	C = C CH₂ twist vibr C-O, C = O str	China/2020	Xu et al. (2020)
Marine and estuarine sediments	45–1000 Fiber, fragment	-	-	USA/2023	El Khatib et al. (2023)
WWTP	25–250 fibers	-	-	Czech Republic/2023	Spacilova et al. (2023)
Sediment of mangroves	0.3–5 mm Fiber, film	860, 1445, 1718, 3076	C–C str CH₂ bending C = O str C-H str	India/2024	Valsan et al. (2024)
Surface water samples	20–500 fibers	-	-	France/2019	Kazour et al. (2019)
Omega-3 oil samples	> 5	-	-	S. Korea/2024	Kim et al. (2024)
Lake samples	20–80	-	-	China/2023	Ma et al. (2023)
Seafood	< 1.6	-	-	France/2016	Dehaut et al. (2016)
Landfill leachate and sludge	Majority less than 50	-	-	China/2020	Sun et al. (2021)
MP samples	-	633, 707, 859, 1096, 1288, 1615, 1721	-	China/2023	Ren et al. (2023)
MP samples	300–1000	1288, 1615, 1721, 3076	-	China/2024	Liu et al. (2024)
Kitchen sponge	< 400 fibers	640, 870, 1110, 1300, 1620, 2910	-	Australia/2022	Lou et al. (2022)
Coastal sea water	> 100	793, 858 1291 1613 1715	Out-of-plane deformation of the aromatic ring C–O–C str, vbr Aromatic bending vbr C = O st. vbr. Ester group	Brazil/2022	Nolasco et al. (2022)

### Polyamide

Table 8 presents data on the analysis of PA MPs using Raman spectroscopy, with samples obtained from various sources. PA is a versatile polymer known for its resistance to heat and chemicals, ease of processing, and widespread industrial use (Feldman 2008). PA fibers, particularly Nylon 6 (NY6) and Nylon 66 (NY66)—the two most commonly produced types—are extensively used in textiles and other applications, primarily differing in their melting points (Feldman 2008). During laundering, PA MPs, especially threads and filaments, are easily released into wastewater and subsequently enter aquatic environments. These MPs can penetrate and accumulate in the tissues of aquatic organisms. Figure S4 displays a representative Raman spectrum of PA (Rytelewska and Dabrowska 2022). Key spectral features include C–CO stretching at 940 cm⁻^1^; C–C stretching near 1085, 1106, and 1126 cm⁻^1^; CH₂ twisting vibrations at 1294 and 1307 cm⁻^1^; CH₂ bending at 1440 cm⁻^1^; CH₂ rocking at 1463 cm⁻^1^; and C–H stretching at 2882 cm⁻^1^ (Kadac-Czapska 2023; Sunil et al. 2024a). A prominent peak at 1634 cm⁻^1^ corresponds to the Amide I band, associated with the C = O stretching vibration of the amide group (–NH–C = O). Additionally, the band at 1270 cm⁻^1^ represents the Amide band, encompassing C–N stretching and N–H bending vibrations (Kadac-Czapska 2023).

**Table 8:** Raman spectral profiles of PA, *NY6, and *NY 66 MPs

Source	Size (µm)	Raman fingerprint (cmˉ^1^)	Band assignment	Country/year	Ref
Fresh water	> 1 Foil	935 1298 1448 2942	-CCO stretching -CN stretching -CH2 bending -CH stretching	Poland/2022	Rytelewska and Dabrowska (2022)
**Marine environment	200	1445, 1636, 2908, 3304	-	China/2022	Liu et al. (2022)
*MP particles	15–20 (average) powder	1130, 1440, 1620, 2850	-	Germany/2020	Sarau et al. (2020)
*Cow milk	> 5	920, 1140, 1300, 1450, 1650		Switzerland/2021	Da Costa Filho et al. (2021)
Deep ocean	> 100 < 5	-	-	USA/2019	Choy et al. (2019)
*Water	< 5 mm	1620	C = O str	India/2022	Chakraborty et al. (2022)
Beach sediment	0.2–5 mm Fiber, fragment	-	-	Germany/2021	Schroder et al. (2021)
**Aquatic system	< 5 mm	1283	C-N str	Italy/2021	Nava et al. (2021)
*Aquatic system	< 5 mm	1636 3302	C = O str Characteristic NH str	Italy/2021	Nava et al. (2021)
Drinking water plants	1–50	1460, 2900	-	Germany/2023	Maurizi et al. (2023)
*Rhine estuary	> 12	-	-	Netherlands/2018	Zada et al. (2018)
*Salt	> 1.55	1309, 1440, 1641, 2920, 3300	-	Bangladesh/2023	Mazumder et al. (2023)
*Tea filter bags	> 1 Fiber, fragments	1081 1130 1443 2904	C–C str C–C sym. Str CNH bending CH₂ str	China/2022	Mei et al. (2022)
Paper cup	1–15 film	1441 1297 1638 2891	C-H bending Amide III Amide I CH₂ asym str	China/2023	Zhang T et al. (2023)
Plastic samples	80–250	-	-	Finland/2024	Hrovat et al. (2024)
WWTP	> 20 fiber	-	-	Finland/2022	Tsering et al. (2022)
Ocean surface water	2.06–10.5	-	-	Japan/2020	Ripken et al. (2021)
Snack boxes	15–180	1002	C–C-C ring bending	China/2022	Jin et al. (2022)
Bivalve tissue	> 50 fibers	1100, 1450, 1630	-	UK/2019	Thiele et al. (2019)
Lagooning sludge	< 500 Fibers, fragments	-	-	Morocco/2020	El Hayany et al. (2020)
Infant formulas	126–4380 Fiber, fragments	1270 1440 2882	C-N str CH₂ bending C-H str	Poland/2023	Kadac-Czapska et al. (2023)
Mussel samples	3–60 fibers	1450, 1640	-	Germany/2020	Kumar et al. (2021)
Marine and estuarine sediments	45–1000 Flake, fragments	-	-	USA/2023	El Khatib et al. (2023)
*WWTP	25–250 fibers	-	-	Czech Republic/2023	Spacilova et al. (2023)
Surface water samples	20–500 Fibers, films	-	-	France/2019	Kazour et al. (2019)
Paddy soil samples	10–50 Irregular shaped	2850–2900	-	S. Korea/2024	Rhee et al. (2024)
Marine environment	< 1 mm Fragment, fibers	1296, 2878, 2902	- C-H(-CH₂) str	India/2022	Sambandam et al. (2022)
**MP samples	300–1000	1440, 2841, 2884	-	China/2024	Liu et al. (2024)
Estuarine, mangrove sediments	-	865, 927, 1058, 1090, 1133, 1280, 1316, 1456, 1619, 2875, 2932, 2970	-	Brazil/2024	Santos et al. (2024)
Kitchen sponge	< 400 fibers	1100, 1310, 1450, 1650, 2910	C–C skeleton str C-H_2_ twisting C-H_2_ bending C = O str C-H str	Australia/2022	Luo et al. (2022b)
Bleached mangrove (environmental) MP samples	< 5 mm	-	-	China/2023	Liu M et al. (2023)

### Polyethylene

Table 9 presents data on PE MPs analyzed using Raman spectroscopy with samples obtained from various sources. PE is one of the most widely used polymers, commonly found in disposable plastic bags, toys, food packaging, bottle caps, containers, and cosmetic products (Rytelewska and Dabrowska 2022). Due to its widespread use and poor disposal practices, PE contributes significantly to the accumulation of plastic waste in the environment (Kazour et al. 2019). The analyzed MP samples included various shapes such as films, pellets, fibers, and fragments. Figure S5 displays the Raman spectrum of PE (Rytelewska and Dabrowska 2022).

**Table 9:** Raman spectral profile of PE MPs

Source	Size (µm)	Raman fingerprint (cmˉ^1^)	Band assignment	Country/year	Ref
Freshwater and WWTP	> 1 Foil, fragment	1200–1500 2800–3200	CH₂ vibration CH₂ str	Poland/2022	Rytelewska and Dabrowska (2022)
Marine environment	> 0.45	1298, 1439, 2889	-	China/2022	Liu et al. (2022)
MP particles	1–106 Clear microspheres, powder	1250, 1440, 2720	-	Germany/2020	Sarau et al. (2020)
Aerosol samples	> 5	1300, 1125, 1450	-	USA/2020	Trainic et al. (2020)
Fish, mussels, and sea water	5.4–15.1 1–5 mm Fragment, film, fibers	1118–1200 1288 1432–1450 2870–2950	C = C Backbone str CH₂ twisting CH₂ bending CH₂ sym-asym str	Greece/2023	Miserli et al. (2023)
Beach sediment	> 500	1061, 1127, 1297, 1448, 2845, 2886	-	Germany/2016	Kappler et al. (2016)
Mussels and pearl oysters	> 0.8 Fibers, fragments	808, 1061, 1127, 1295, 2849, 2884	-	Greece/2023	Manolaki et al. (2023)
Human	30–1800 fragments	1080, 1140, 1310, 1460	-	Hong Kong/2022	Ho et al. (2022)
Human breastmilk	2–12 µm Transparent sphere	600, 1060, 1300, 1440	-	Italy/2022	Ragusa et al. (2022)
Mediterranean Sea	1.2–3 µm Fragment, film	1150, 1300, 2850	-	Poland/2023	Dabrowska et al. (2023)
Dried fish	1–1000 Film, fragment	-	-	Malaysia/2017	Karami et al. (2017)
Fish meal	< 100 Fragment, film	800, 1250, 1500, 1700, 2280	-	UK/2021	Thiele et al. (2021)
Cow milk	> 5	1050, 1140, 1300	-	Switzerland/2021	Da Costa Filho et al. (2021)
Water	< 5 mm	1450	CH₂ sym	India/2022	Chakraborty et al. (2022)
MP sample	< 10	2850	-	USA/2020	Primpke et al. (2020)
Wastewater sludge	Particle fibers	-	-	Finland/2021	Asamoah et al. (2021)
MP samples	-	1100, 1270, 1450, 2850	-	Belgium/2024	Plazas et al. (2024)
Beach sediment	0.2–5 mm Fiber, fragments	-	-	Germany/2021	Schroder et al. (2021)
Marine sediment	45–1000 fiber, fragment, sphere	-	-	USA/2022	Cashman et al. (2022)
Aquatic system	< 5 mm	1295 2850 2883	CH₂ twisting Sym CH₂ Asym CH₂	Italy/2021	Nava et al. (2021)
Sea sediment	0.2–5 mm Fiber, fragments, film	1060, 1130, 1290, 1420, 1440	-	Russia/2020	Esiukova et al. (2020)
Stormwater drainage systems	25–530 Fragments, fibers	2820	-	Australia/2022	Bond et al. (2022)
Rhine estuary	> 12	-	-	Netherlands/2018	Zada et al. (2018)
Air sample	20–850 Fiber, fragments	1063, 1150, 1294	-	USA/2020	Gaston et al. (2020)
Tea filter bag	> 1 Fiber, fragments	1063 1130 1441 2885	Asym. C–C str C–C str CH₂ bending Asym CH₂ str	China/2022	Mei et al. (2022)
PE pellets and bottle caps	1–3 small ball 50–100 fragment	1415, 2850	-	Ireland/2022	Li et al. (2022)
Crater lake	8–15 Fragment	1171, 1327, 1460, 2900, 3058	-	Turkey/2020	Comakl1 et al. (2020)
PM10 aerosol	> 10	1400, 1280, 2850	-	S. Korea/2023	Yoo et al. (2023)
DWTP	1–100 Fibers, fragment	-	-	Iran/2021	Adib et al. (2021)
MP samples	39–155	1292, 1430	-	China/2021	Yin et al. (2021)
Groundwater	> 20 Fibers, fragment	1062 1296 1440 2850, 2884	C–C anti. Sym vibr Anti.sym.CH₂ bending CH₂ sym.bending CH₂ sym. Vibration	Greece/2024	Perraki et al. (2024)
River, freshwater	1–5 mm Fragment, fiber	1064, 1297, 1443, 2778	-	Romania/2024	Gheorghe et al. (2024)
Marie environment	> 12	-	-	France/2024	Grand et al. (2024)
Paper cup	1–60 film	1064 1297 1440 2880	C–C str CH₂ twisting Sym CH₂ Asym CH₂ str	China/2023	Zhang T et al. (2023)
Human kidneys and urine	3–13 Fragment	1290, 1480, 2880	-	Italy/2024	Massardo et al. (2024)
Sea salt	30–150/fiber	-	-	UK/2023	Thiele et al. (2023)
Plastic tap	20–300	1490, 2900	-	Poland/2021	Dabrowska (2021)
Recycled plastic samples	80–250	-	-	Finland/2024	Hrovat et al. (2024)
WWTP	> 20 fragment	-	-	Finland/2022	Tsering et al. (2022)
Ocean surface water	1.40–30.5	1058 1286 1429	C–C sym str CH₂ twisting CH₂ sym	Japan/2020	Ripken et al. (2021)
Cosmetics sample	1–400 microbeads	1450 2880	CH₂ bending CH₂ asym.band	Poland/2022	Dabrowska et al. (2022)
Water bottle	200 nm	2880	-	Italy/2022	Winkler et al. (2022)
Water in glass and beverage cartoon	5–50	-	-	Germany/2018	Schymanksi et al. (2018)
IPWWTP	> 10	-	-	Germany/2023	Barkmann-Metaj et al. (2023)
Ocean	< 50	-	-	USA/2024	Faull et al. (2024)
Beach pellet samples	< 5 mm	1060 1293 2890	C–C str CH₂ wagging CH-str	Italy/2024	Donato et al. (2024)
Odontocetes species	1–3 mm	447, 1296	-	Spain/2021	Montoto-Martinez et al. (2021)
Iceland sediments	> 150 fibers	-	-	Ireland/2021	Loughlin et al. (2021)
Sea	0.335–5 mm Pellet, fragment, film	1060, 1140, 1300	-	France/2016	Frere et al. (2016)
Small MP samples	90–106 Particles	3000∼2800	-	Japan/2023	Yang et al. (2023)
Water bottle cap	20–100	-	-	Italy/2023	Gambino et al. (2023)
Lagooning sludge	< 500 Fibers, fragments	-	-	Morocco/2020	El Hayany et al. (2020)
Infant formula	126–4380 Fibers, fragment	1063, 1124 1416, 1430 2843	C–C str CH₂ bending Sym.CH₃ str	Poland/2023	Kadac-Czapska et al. (2023)
Mussel samples	3–60 Fibers, fragments	1050, 1295, 1420	-	Germany/2020	Kumar et al. (2021)
Beach and sea water	15–900 Fibers	1060, 1126 1294, 1438	C–C str CH₂ twisting CH₂ bending	India/2024	Sunil et al. (2024a)
Water environments	10–25–48–150–270–500 µm particles	1064 1132 1293 1450	C–C str C–C str CH₂ twisting CH₂ bending	China/2024	Luo et al. (2024)
Marine and estuarine sediments	45–1000 fragment, microbeads	-	-	USA/2023	El Khatib et al. (2023)
MP sample	1–4 Powder microspheres	1060 1291 2872	Anti.sym. C–C str CH₂ twisting Anti.sym. CH₂ str	Hungary/2022	Mikac et al. (2023)
WWTP	25–250 Flakes	-	-	Czech Republic/2023	Spacilova et al. (2023)
Sediment of mangroves	0.3–5 mm Fiber, films	1292 1445 2880 2845	CH₂ twisting Sym CH₂ deformtn CH₂ Asym. str CH₂ sym str	India/2024	Valsan et al. (2024)
MP samples	4.2 Powder	1060, 1130, 1300, 2850	-	France/2024	Ompala et al. (2024)
Sludge and effluent samples	> 200 Fibers, fragments	-	-	France/2019	Kazour et al. (2019)
Paddy soil samples	27–32 Spherical beads	2850–2900	-	S. Korea/2023	Rhee et al. (2024)
MPs + natural organic matter	34–50 powder	1062, 1295, 1440, 2844, 2878	-	S. Korea/2023	Lee S et al. (2023)
Omega-3 oil samples	> 5	1127 1293 1417, 1439, 1465 2849, 2883	C–C str CH₂ twisting CH₂ bending C-H str	S. Korea/2024	Kim et al. (2024)
Lake samples	20–80	-	-	China/2023	Ma et al. (2023)
Marine environment	< 1 mm Fibers, fragments	1070 1175	C–C str CH₂ rocking	India/2022	Sambandam et al. (2022)
Landfill leachate and sludge	< 100	-	-	China/2020	Sun et al. (2021)

High-density polyethylene (HDPE, Table 10) and LDPE (Table 11) share the same carbon backbone (Unnimaya et al. 2023). In their Raman spectra (Figures S6-S8), C–C bonds in the trans-conformation show asymmetric stretching near 1063 cm⁻^1^ and symmetric C–C stretching around 1129 cm⁻^1^. Distinct CH₂ peaks are observed in the range of 1285–1294 cm⁻^1^ (CH₂ twisting), along with CH₂ deformation bands at 1416 cm⁻^1^, 1430 cm⁻^1^, and 1452 cm⁻^1^. Additionally, CH₂ symmetric and asymmetric stretching bands appear at approximately 2846 cm⁻^1^ and 2875–2881 cm⁻^1^, respectively (Kadac-Czapska et al*.* 2023). Raman spectroscopy effectively distinguishes LDPE from HDPE due to its sensitivity to differences in crystallinity (Miserli et al. 2023; Saad et al. 2022; Unnimaya et al. 2023). Although the Raman spectra of LDPE and HDPE are similar (see Figure S8), they can be differentiated based on subtle variations, particularly in the C–H stretching region (Unnimaya et al. 2023).

**Table 10:** Raman spectral profile of HDPE MPs

Source	Size (µm)	Raman fingerprint (cmˉ^1^)	Band assignment	Country/year	Ref
Plastic consumables	39–538	1059 1167 2847 2879	CH bend. C–C str C–C str Sym. CH₂ str Asym. CH₂ str	India/2023	Unnimaya et al. (2023)
Seawater	1–5 mm	1050, 1120, 1290, 1400, 2850	-	Greece/2023	Miserli et al. (2023)
Plastic samples	80–250	-	-	Finland/2024	Hrovat et al. (2024)
Water bottle Cap	200 nm	1460 2950	CH₂ bent CH₂ str	Italy/2022	Winkler et al. (2022)
Fish	0.04–4.94 mm Fibers, fragments	1130, 1300, 1450, 2870	-	S. Africa/2024	Saad and Alamin (2024)
River/fresh water	0.5–5 mm Fiber, fragment	1050, 1120, 1300, 1450, 2850, 2880	-	S. Africa/2022	Saad et al. (2022)
MP sample	< 5 mm	1060 1132 1290	Characteristic peak C–C vbr CH₂ oscillating vbr	China/2022	Xue et al. (2022)
Seafood	< 1.6	-	-	France/2016	Dehaut et al. (2016)
River water	Mean 2.12 ± 0.02	-	-	Spain/2023	Trujillo et al. (2023)

**Table 11:** Raman spectral profile of LDPE MPs

Source	Size (µm)	Raman fingerprint (cmˉ^1^)	Band assignment	Country/year	Reference
Plastic consumables	39–538	1059 1292 1436 2847	CH bend. C–C str CH₂ twisting CH₂ bending Sym. CH₂ str	India/2023	Unnimaya et al. (2023)
Fish, mussels, seawater	5.4–15.1 1–5 mm	1060, 1130, 1300, 1450, 2850, 2900	-	Greece/2023	Miserli et al. (2023)
Lichens	45–796 Fibers, fragments	1330, 1450, 2880	-	Italy/2021	Loppi et al. (2021)
Salt	> 1.55	1060, 1130, 1296, 1441	-	Bangladesh/2023	Mazumder et al. (2023)
WWTP	< 100 fragment	1060, 1140, 1300, 1450	-	Canada/2021	Lavoy and Crossman (2021)
LDPE MP samples	-	1063 1129 1294 1440	Asym.C–C str Sym. C–C str CH₂ twisting CH₂ bending vibr	China/2023	Ren et al. (2023)
MP sample in water and air	> 100 pellet	1063 1129 1440 2850	Asym.C–C str Sym. C–C str CH₂ bending Sym. C–C str	USA/2022	Phan et al. (2022)
Plastic samples	80–250	1290, 1460, 2880	-	Finland/2024	Hrovat et al. (2024)
Greenhouse coversheets	1–25 µm and 200 nm films	1064, 1086 1442, 1460 2848	C–C str CH₂ bending Sym. C-H str	Spain/2024	Sorasan et al. (2024)
Bivalve tissue	> 50 film	1300, 2900	-	UK/2019	Thiele et al. (2019)
River/fresh water	0.5–5 mm Fiber, fragment	1150, 1300, 1450, 2850, 2900	-	S. Africa/2022	Saad et al. (2022)
Seafood	< 1.6	-	-	France/2016	Dehaut et al. (2016)
MP samples	-	1063, 1129, 1173, 1294, 1419, 1440	-	China/2023	Ren et al. (2023)
MP samples	300–1000	1296, 1440, 2849, 2884	-	China/2024	Liu et al. (2024)
Photodegraded samples from the Atlantic Ocean	≥ 10 μm particles	1063, 1130, 1296, 1418, 1441, 1463	CH₂ wagging, CH₂ deformation, CH₂ bending	Denmark/2015	Lenz et al. (2015)

### Polypropylene

Table 12 presents data related to PP analysis using Raman spectroscopy, with samples collected from various sources. PP is widely used due to its durability and low density and is found in a range of products including packaging, labeling materials, textiles, stationery, plastic parts, reusable containers, laboratory equipment, and automotive components (Phan et al. 2022; Rytelewska and Dabrowska 2022). In environmental samples, PP MPs are commonly detected as fibers and fragments (Bottari et al. 2022; Phan et al. 2022). Figure S9 shows the Raman spectrum of PP (Rytelewska and Dabrowska 2022), which includes several characteristic peaks useful for identification. Notable bands include CH₂ wagging and CH bending at 397 cm⁻^1^; CH₂ rocking, C–C stretching, and C–CH₃ stretching at 807 cm⁻^1^; and C–C stretching and CH₂ rocking at 839 cm⁻^1^. Additional peaks are observed at 946 cm⁻^1^ (CH₃ rocking), 971 cm⁻^1^ (C–C stretching), and 1035 cm⁻^1^ (C–CH₃ stretching, C–C stretching, and C–H bending). The spectrum also includes bands at 1150 cm⁻^1^ (C–C stretching and CH₃ bending), 1217 cm⁻^1^ (CH₂ twisting, CH bending, and C–C stretching), and 1305 cm⁻^1^ (CH₂ twisting). Peaks at 1327 cm⁻^1^ correspond to CH stretching, CH₂ wagging, and CH₃ bending, while 1458 cm⁻^1^ indicates CH₂ bending and CH₃ asymmetric bending. The CH₂ stretching band appears at 2811 cm⁻^1^, and the symmetric and asymmetric CH₃ stretching vibrations are observed at 2885 cm⁻^1^ and 2954–2963 cm⁻^1^, respectively (Perraki et al. 2024; Sunil et al. 2024a).

**Table 12:** Raman spectral profile of PP MPs

**Source**	**Size (µm)**	**Raman fingerprint (cmˉ**^**1**^**)**	**Band assignment**	**Country/year**	**Ref**
Freshwater and WWTP	> 1 fragment	1125, 1200, 1490, 2880	-	Poland/2022	Rytelewska and Dabrowska (2022)
Beach sand	0.45	815, 2895	-	China/2022	Liu et al. (2022)
Aerosol samples	> 5	800, 850, 1150, 1450	-	USA/2020	Trainic et al. (2020)
Seawater	1–5 mm	800, 920, 1150, 1350, 1440, 2950	-	Greece/2023	Miserli et al. (2023)
Beach sediment	< 400	Spectral range of 2780–2980	-	Germany/2016	Kappler et al. (2016)
Mussels and pearl oysters	> 0.8 Fibers, fragments	842, 972, 1165, 1440, 2884	-	Greece/2023	Manolaki et al. (2023)
Human	30–1800 fragments	810, 860, 980, 1160, 1180, 1340, 1480	-	Hong Kong/2022	Ho et al. (2022)
Human breastmilk	2–12 µm irregular fragment	850, 1340, 1450	-	Italy/2022	Ragusa et al. (2022)
Mediterranean Sea	1.2–3 µm Fragment	850, 1200, 1375, 1490, 2850	-	Poland/2023	Dabrowska et al. (2023)
Fish	0.4–4.2 µm Fibers, fragment	-	-	Italy/2022	Bottari et al. (2022)
Dried fish	1–1000 Film, fragment	-	-	Malaysia/2017	Karami et al. (2017)
Fish meal	< 100 microfibers	-	-	UK/2021	Thiele et al. (2021)
Cow milk	> 5	810, 850, 960, 1150, 1340, 1450	-	Switzerland/2021	Da Costa Filho et al. (2021)
Water	< 5 mm	1500	CH₃ sym. Bend	India/2022	Chakraborty et al. (2022)
Wastewater sludge	Particle fibers	-	-	Finland/2021	Asamoah et al. (2021)
MP samples	-	800, 950, 1250, 1350, 1500, 2950	-	Belgium/2024	Plazas et al. (2024)
Beach sediment	0.2–5 mm Fiber, fragment	-	-	Germany/2021	Schroder et al. (2021)
Marine sediment	40–1000 fiber, fragment, sphere	-	-	USA/2022	Cashman et al. (2022)
Aquatic system	< 5 mm	1330 1458 2883	CH-bending Asym. CH₃ bending Symmt.str. CH₃	Italy/2021	Nava et al. (2021)
Drinking water plants	1–50	1450, 2880	-	Germany/2023	Maurizi et al. (2023)
Sea sediment	0.2–5 mm Fibers, fragments, films	810, 840, 970, 1160, 1330, 1450	-	Russia/2020	Esiukova et al. (2020)
Stormwater drainage systems	25–530 Fragments, fibers	708 2905	-	Australia/2022	Bond et al. (2022)
Rhine estuary	> 12	-	-	Netherlands/2018	Zada et al. (2018)
Salt	> 1.55	841, 976, 1155, 1462, 2886	-	Bangladesh/2023	Mazumder et al. (2023)
Tea filter bags	> 1 Fiber, fragments	811 1332 1463 2886	CH₃ str CH₃ rocking CH₂ bending CH₃ sym. Str	China/2022	Mei et al. (2022)
Tap water	100 nm	1435–1460 2780–2980	-CH₂ bending -CH₂-CH₃ str. Vibr	Norway/2023	Lou et al. (2023)
Jellyfish	< 300	-	-	Switzerland/2023	Caldwell et al. (2021)
Food containers	50–100 fragments	2800–3000	-	Ireland/2022	Li et al. (2022)
Crater lake	8–15 fragment	840, 943, 1185, 1455, 2935, 2966	-	Turkey/2020	Comakl1 et al. (2020)
DWTP	1–100 Fiber, fragment	-	-	Iran/2021	Adib et al. (2021)
MP samples	39–155	810, 1150, 1450	-	China/2021	Yin et al. (2021)
Groundwater	> 20 Fibers, fragment	809 1330 1460 2888	CH₂ vibration CH vibration CH₃ Asym.bending CH₂ str	Greece/2024	Perraki et al. (2024)
River, freshwater	1–5 mm Fiber, fragment	1378, 1880, 2465	-	Romania/2024	Gheorghe et al. (2024)
Marine environment	> 12	-	-	France/2024	Grand et al. (2024)
Tiger sharks GI tracts	45–355 fibers	810, 1340, 1450	-	USA/2024	Munno et al. (2024)
Lake water and sediment	> 11 fibers	-	-	Finland/2022	Viitala et al. (2022)
WWTP	< 100 Fiber-fragment	800, 960, 1150, 1350, 1460	-	Canada/2021	Lavoy and Crossman (2021)
Coastal sediments	10–20 fiber	1445, 2855	-	S. Arabia/2020	Laptenok et al. (2020)
Sea salt	30–150/fiber	-	-	UK/2023	Thiele et al. (2023)
MP sample in water and air	> 100 pellet	808 1153 1460 2960	CH₂ rock, C–C str C–C str Asym. CH₃ bent Asym CH str	USA/2022	Phan et al. (2022)
Plastic samples	80–250	-	-	Finland/2024	Hrovat et al. (2024)
Marine environment	170–410 fiber	2950	-	S.Arabia/2023	Genchi et al. (2023)
WWTP	> 20 fiber	-	-	Finland/2022	Tsering et al. (2022)
Ocean surface water	3.07–6.15	950, 1150, 1350, 1460	-	Japan/2021	Ripken et al. (2021)
Water in beverage cartoon	5–50	-	-	Germany/2018	Schymanski et al. (2017)
IPWWTP	> 10	-	-	Germany/2023	Barkmann-Metaj et al. (2023)
Juice boxes	15–180	1459 2884	Asym. CH₃ bending Symmt.str. CH₂	China/2022	Jin et al. (2022)
Ocean	< 50	-	-	USA/2024	Faull (2024)
Beach pellet samples	< 5 mm	811 1460 2884	C–CH₃ str CH₂ bending CH₃ str	Italy/2024	Donato et al. (2024)
Odontocetes species	1–3 mm	1350, 1550	-	Spain/2021	Montoto-Martinez et al. (2021)
Bivalve tissue	> 50 fibers, fragments	800, 1490	-	UK/2019	Thiele et al. (2019)
Sea	0.335–5 mm Pellet, fragment, film	805, 1450	-	France/2016	Frere et al. (2016)
Lagooning sludge	< 500 Fiber, fragments	-	-	Morocco/2020	El Hayany et al. (2020)
Fish	0.04–4.94 mm Fibers, fragment	800, 1320, 1450, 2900	-	S. Africa/2024	Saad and Alamin (2024)
MP sample	Fragment	-	-	UK/2023	Kukkola et al. (2023)
Infant formula	126–4380 Fibers, fragment	782, 821 1305, 1330 2811	C–C str CH₂ twisting CH₂ str	Poland/2024	Kadac-Czapska et al. (2023)
Mussel samples	3–60 Fiber, fragments	1150, 1325, 1450	-	Germany/2020	Kumar et al. (2021)
River/fresh water	0.5–5 mm Fiber, fragment	800, 1150, 1460, 2850, 2900, 2970	-	S. Africa/2022	Saad et al. (2022)
MP sample	< 5 mm	805, 970, 1152, 1456	-	China/2022	Xue et al. (2022)
Marine and estuarine sediments	45–1000 Fiber, fragment	-	-	USA/2023	El Khatib et al. (2023)
WWTP	25–250 Flakes	-	-	Czech Republic/2023	Spacilova et al. (2023)
Sediment of mangroves	0.3–5 mm Fiber, film	1147 1456 2885, 2958	C-H str CH₃ Asym. Bending CH₃ Sym.-Asym str	India/2024	Valsan et al. (2024)
Mouse liver	10–120 Irregular granule	811 1330 1459 2884	CH bending, CH₂ twisting CH₃ asym. Bend	China/2023	Liu M et al. (2023)
MP samples	10 powder	-	-	France/2024	Ompala et al. (2024)
Marine environment	> 200 Fiber, fragments	-	-	France/2019	Kazour et al. (2019)
Omega-3 oil samples	> 5	-	-	S. Korea/2024	Kim et al. (2024)
Marine environment	< 1 mm Fibers, fragments	1154, 1331, 1462, 2720, 2844 cm¯^1^		India/2022	Sambandam et al. (2022)
Seafood	< 1.6	-	-	France/2016	Dehaut et al. (2016)
Landfill leachate and sludge	< 100	-	-	China/2021	Sun et al. (2021)
MP samples	-	811, 842, 977, 1155, 1330, 1462		China/2023	Ren et al. (2023)
MP samples	300–1000	811, 830, 1330 1462, 2884, 2956	-	China/2024	Liu et al. (2024)
Coastal seawater	1.66 mm fiber	809, 1152, 1170, 1434, 1458, 2780, 2980	C–C stretching C–C stretching C-H₃ rocking C-H bending C-H₂ and C-H₃ group vibration	Brazil, 2022	Nolasco et al. (2022)

### Polyvinyl chloride

Table 13 presents data related to PVC analysis using Raman spectroscopy, based on samples from various sources. PVC is a durable and cost-effective polymer widely used in the production of construction and building materials, water pipes, children’s toys, packaging, and healthcare products (Seewoo et al. 2024). Although recyclable, PVC waste tends to persist in the environment for extended periods, contributing to the accumulation of MPs. PVC MPs are predominantly detected in aquatic environments, but they have also been found in the human body, including in breast milk (Ho et al. 2022; Ragusa et al. 2022). Figure S10 displays the Raman spectrum of PVC (Ragusa et al. 2022). Characteristic peaks include C–Cl in-plane bending in *trans*-HClC = CHCl at 361 cm⁻^1^, C–Cl stretching in Cl₂C = CHCl at 636 cm⁻^1^, and C–Cl asymmetric stretching in H₂C = CHCl at 695 cm⁻^1^. Additional peaks appear at 1179 cm⁻^1^ (CH₂ twisting) and at 1431 cm⁻^1^ and 2914 cm⁻^1^, both corresponding to CH₂ bending (Liu et al. 2022; Sunil et al. 2024a).

**Table 13:** Raman spectral profile of PVC MPs

**Source**	**Size (µm)**	**Raman fingerprint (cmˉ**^**1**^**)**	**Band assignment**	**Country/year**	**Ref**
MP particles	< 50 powder	630, 2880	-	Germany/2020	Sarau et al. (2020)
Beach sediment	< 400	Spectral range of 2780–2980	-	Germany/2016	Kappler et al. (2016)
Human	30–1800 fragments	700, 1180, 1440	-	Hong Kong/2022	Ho et al. (2022)
Human breastmilk	2–12 µm irregular fragment	650, 700, 1300, 1400–1600	-	Italy/2022	Ragusa et al. (2022)
Deep ocean	> 100 < 5	-	-	USA/2019	Choy et al. (2019)
Water	< 5 mm	510	C–Cl str	India/2022	Chakraborty (2022)
MP sample	< 10	2900	-	USA/2020	Primpke et al. (2020)
MP samples	-	650, 1490, 2900	-	Belgium/2024	Plazas et al. (2024)
Marine sediment	40–1000 fiber, fragment, sphere	-	-	USA/2022	Cashman et al. (2022)
Aquatic system	< 5 mm	638 2914	C–Cl str. vibration Asymm. CH₂ str	Italy/2021	Nava et al. (2021)
Drinking water plants	1–50	2915	-	Germany/2023	Maurizi et al. (2023)
Air sample	20–850 fiber, fragment	-	-	USA/2020	Gaston et al. (2020)
MP samples	39–155	625, 686, 1420	-	China/2021	Yin et al. (2021)
Plastic samples	80–250	-	-	Finland/2024	Hrovat et al. (2024)
WWTP	> 20 fragment	-	-	Finland/2022	Tsering et al. (2022)
Ocean surface water	8.97–47.8	610 1325 1724	C–Cl str CH₂ twisting Ester CO str	Japan/2021	Ripken et al. (2021)
IPWWTP	> 10	-	-	Germany/2023	Barkmann-Metaj et al. (2023)
Medicine vials	15–180	636 1431 2914	C–Cl str C-H str C-H str	China/2022	Jin et al. (2022)
Iceland sediments	> 150 fibers	-	-	Ireland/2021	Loughlin et al. (2021)
Bivalve tissue	> 50 film	620, 1050	-	UK/2019	Thiele et al. (2019)
MP sample	Fragment	-	-	UK/2023	Kukkola et al. (2023)
Marine and estuarine sediments	45–1000 foam, fragment	-	-	USA/2023	El Khatib et al. (2023)
Lake samples	20–80	-	-	China/2023	Ma et al. (2023)
Marine environment	< 1 mm Fragment, films	1462, 1525, 2436	-	India/2022	Sambandam et al. (2022)
Seafood	< 1.6	-	-	France/2016	Dehaut et al. (2016)
MP samples	300–1000	637, 707, 1431, 2884	-	China/2024	Liu et al. (2024)
Human placenta	1.03–6.84	-	-	China/2024	Yun et al. (2024)
Airborne lab MPs	1–1000 fibers	-	-	UK/2024	Bhat et al. (2024)
Bleached mangrove (environmental) MP samples	< 5 mm	-	-	China/2023	Liu Y et al. (2023)
Photo-degraded samples from the Atlantic Ocean	≥ 10 μm particles	693, 637, 2916	C–Cl bonds C-H stretching vibrations	Denmark/2015	Lenz et al. (2015)

### Polydimethylsiloxane

The Raman spectral profile of PDMS is presented in Table 14. PDMS is a transparent, highly hydrophobic, and chemically resistant silicone that does not bioaccumulate. Its high thermal stability, corrosion resistance, biocompatibility, flexibility, ease of use, low production cost, gas permeability, and hyper-elasticity make it widely used in a range of products, including polishes, detergents, personal care items, foods, pharmaceuticals, paints, plastic bags, elastomers, lenses, biomedical devices, synthetic fibers, adhesive membranes for pervaporation and filtration, and sensors such as those used in gas chromatography (Seethapathy and Gorecki 2012). Raman spectroscopy has been used to identify and characterize aerosol samples containing PDMS collected over the North Atlantic Ocean (Ariati et al. 2021; Seethapathy and Gorecki 2012; Trainic et al. 2020). Airborne PDMS MPs larger than 5 µm were identified by their characteristic peaks at 495 cm⁻^1^, 700 cm⁻^1^, and 1100 cm⁻^1^ (Trainic et al. 2020). Figure S11 shows the characteristic Raman spectrum of PDMS, which includes asymmetric stretching vibrations of Si–CH₃ groups at 1254 cm⁻^1^, asymmetric bending deformation modes of CH₃ groups at 1417–1435 cm⁻^1^, and C–H stretching modes of methyl groups bonded to silicon atoms at 2899 cm⁻^1^ (Durdakvova et al*.* 2020).

**Table 14:** Raman spectral profile of PDMS MPs

**Source**	**Size (µm)**	**Raman fingerprint (cmˉ**^**1**^**)**	**Country/year**	**Ref**
Aerosol samples	> 5	495, 700, 1100	USA/2020	Trainic et al. (2020)
Sea sediments	0.2–5 mm Fibers, films, fragments	690, 1000	Russia/2020	Esiukova et al. (2020)
Medical syringes (solid and liquid PDMS)	< ~ 25 nm ∼210 particle/mm^2^	2910	Australia/2024	Fang et al. (2024)
De-ionized water solution	10	~ 800, ~ 1000	France/2021	Elsayed et al. (2021)
Kassel wastewater	< 11	-	Germany/2021	Kernchen et al. (2021)

### Polycarbonate

The Raman spectral profile of PC is presented in Table 15. PC is a highly ductile, lightweight engineering polymer known for its excellent thermal and dimensional stability, high flame retardancy (compared to other plastics), optical transparency, superior compressive and tensile strength, strong shear-loading performance, and good melt flowability. It is widely used in the automotive and aerospace industries, especially for car headlight lenses and aircraft windshields. Due to its high strength-to-weight ratio and optical properties, PC is also commonly used in structural components and eyeglass lenses (Seewoo et al. 2024; Subramaniam et al. 2023; Zou et al. 2025). Figure S12 displays the Raman spectrum of PC. The characteristic Raman peaks of PC include benzene ring vibrations at 576 cm⁻^1^, 637 cm⁻^1^, and 826 cm⁻^1^; C–H out-of-plane bending at 705 cm⁻^1^ and 735 cm⁻^1^; O–C(O)–O stretching at 887 cm⁻^1^; C–H in-plane bending at 1110 cm⁻^1^; C–O–C stretching at 1179 cm⁻^1^ and 1236 cm⁻^1^; CH₃ deformation at 1461 cm⁻^1^; phenyl ring vibration at 1590 cm⁻^1^; benzene ring stretching at 1604 cm⁻^1^; and C–H stretching modes in the 2800–3100 cm⁻^1^ region (Sunil et al. 2024a; Xue et al. 2022).

**Table 15:** Raman spectral profile of PC MPs

**Source**	**Size (µm)**	**Raman fingerprint (cmˉ**^**1**^**)**	**Band assignment**	**Country/year**	**Ref**
Freshwater	> 1 Foil, fragments	500, 750, 1490, 1510, 2500, 300	-	Poland/2022	Rytelewska and Dabrowska (2022)
Beach sediment	< 400	Spectral range of 1580–1640	-	Germany/2016	Kappler et al. (2016)
Human breastmilk	2–12 µm Irregular fragment	750, 900, 1250, 1611	-	Italy/2022	Ragusa et al. (2022)
Deep ocean	> 100 < 5	-	-	USA/2019	Choy et al. (2019)
MP samples	39–155	890, 1105	-	China/2021	Yin et al. (2021)
MP sample	< 5 mm	637 827 1110 1233	C atoms str Asym str.O-C-O Str. C chain Asym str.C	China/2022	Xue et al. (2022)
MP sample	15–180	637, 707, 887, 1108, 1176, 1230, 1590	Phenyl ring vibrations C-H out-of-plane bending O-C(O)-O stretching C–O–C stretching	China/2023	Jin et al. (2022)
River/ocean shore sediments	0.063–5 mm	-	-	Italy/2023	Cofano et al. (2023)
MP samples	-	637, 707, 887, 1108, 1230, 1615	-	China/2023	Ren et al. (2023)
MP samples	300–1000	637, 887, 1108, 1176, 1230, 1615, 2881, 2978, 3081	-	China/2024	Liu et al. (2024)
Human placenta	1.03–6.84	-	-	China/2024	Yun et al. (2024)

### Polytetrafluoroethylene

Table 16 presents the Raman spectral profile of PTFE. Commonly known as Teflon, PTFE is highly resistant to corrosion, provides excellent electrical insulation, and exhibits high thermal stability due to the strength of its carbon–fluorine bonds (Furuta et al. 2017). It is widely used in industrial applications such as wire and cable coatings, gaskets, and components in the aviation sector. Its non-stick properties also make it ideal for household cookware, including pots and pans (Furuta et al. 2017). Additionally, PTFE is employed in biomedical applications, including artificial blood vessels, lung membranes, and cosmetic surgery (Furuta et al. 2017). Despite its durability, PTFE MPs of various sizes have been detected in water and marine environments (Bond et al. 2022; Chakraborty 2022; Faull et al. 2024; Kappler et al*.* 2016). Figure S13 shows the Raman spectrum of PTFE. The polymer’s carbon–fluorine backbone gives rise to characteristic Raman peaks primarily associated with C–F and C–C bonds. These include CF₂ stretching vibrations at 732 cm⁻^1^, CF₂ twisting at 1218 cm⁻^1^, symmetric CF₂ stretching at 1302 cm⁻^1^, and asymmetric CF₂ stretching at 1381 cm⁻^1^ (Lee S et al*.* 2023).

**Table 16:** Raman spectral profile of PTFE MPs

**Source**	**Size (µm)**	**Raman fingerprint (cmˉ**^**1**^**)**	**Band assignment**	**Country/year**	**Ref**
Beach sediment	< 400	Spectral range of 709–759 cmˉ^1^	-	Germany/2016	Kappler et al. (2016)
Water	< 5 mm	735	CF₂ symt.str	India/2022	Chakraborty et al. (2022)
Stormwater drainage systems	25–530 Fragments, fibers	399, 809, 974, 1170, 1330	-	Australia/2022	Bond et al. (2022)
Ocean	< 50	-	-	USA/2024	Faull et al. (2024)
MPs + natural organic matter	35 powders	732, 1218, 1381	-	S. Korea/2023	Lee S et al. (2023)
Seafood	< 1.6	-	-	France/2016	Dehaut et al. (2016)
Saline, green bottle fly Lucilia feces, Blatella germanica cockroach	1.32–65.35 nm	Saline (290, 383, 575, 732, 1215, 1297, 1379) Fly feces (572, 599, 732) Cockroach (293, 574, 732, 876, 100, 1092, 1298, 1379, 1597)	CF_2_ mode	Russia/2021	Karpenko et al. ( 2021)
Cooking pot surface	-	290, 385, 730, 1300, 1380	-	Australia/2022	Luo et al. (2022a)
Human placenta	1.03–6.84	-	-	China/2024	Yun et al. (2024)
Marine sediments	> 0.45	-	-	New Zealand/2023	Ribo et al. (2023)
Airborne lab MPs	1–1000 fibers	-	-	UK/2024	Bhat et al. (2024)
MP sample in aqueous solution	< 400	~ 290, ~ 986, ~ 732, ~ 1300	-CF_2_- groups	China/2025	Pu et al. (2025)

### Polymethylmethacrylate

Table 17 presents the Raman spectral profile of PMMA. Also known as acrylic or plexiglass, PMMA is a transparent, lightweight, durable, and biocompatible acrylate polymer with excellent UV resistance. Patented by Otto Röhm in 1928 under the name Plexiglas, some of its earliest applications included aircraft glazing in 1933 and prosthetics in 1936 (Feretis et al. 2003). Today, PMMA is widely used in cosmetics, prosthetics, and as a substitute for glass in various applications (Feretis et al*.* 2023). PMMA MPs have been detected using CARS spectroscopy without requiring chemical pretreatment of soil (Derksen et al. 2020; Rhee et al. 2024). Figure S14 shows the Raman spectrum of PMMA. Key characteristic peaks include C–C–O stretching at 600 cm⁻^1^, C–O–C symmetric stretching at 810 cm⁻^1^, C–C stretching at 968 cm⁻^1^, C–H bending at 1449 cm⁻^1^, C = O stretching at 1727 cm⁻^1^, C–H stretching of O–CH₃ at 2950 cm⁻^1^, and asymmetric CH₂ stretching vibrations (Lee S et al*.* 2023).

**Table 17:** Raman spectral profile of PMMA MPs

**Source**	**Size (µm)**	**Raman fingerprint (cmˉ**^**1**^**)**	**Band assignment**	**Country/year**	**Ref**
Cow milk	> 5	810, 1450, 1710	-	Switzerland/2021	Da Costa Filho et al. (2021)
Water	< 5 mm	814 1700 2957	C = O C–O–C C-H	India/2022	Chakraborty et al. (2022)
Water and sediment	5	622 817 1000 1452 1723	C–C-O str C–O–C symm.str C–C str C-H bending C = O str	USA/2021	Kundu et al. (2021)
WWTP	< 100 fragment	810, 1460, 1730	-	Canada/2021	Lavoy and Crossman (2021)
Water	10 beads	2955	C-H str	Portugal/2018	Araujo et al. (2018)
Mussel samples	3–60 fiber, fragments	1425, 1550	-	Germany/2021	Kumar et al. (2021)
PMMA Beads	2–5 spheres	622 817 1452	C–C-O str C–O–C str C-H bending	China/2020	Xu et al. (2020)
Paddy soil samples	6–50 Spherical beads	2960	-	S. Korea/2023	Rhee et al. (2024)
MPs + natural organic matter	145 Powder	600, 810, 1449, 1727, 2950	-	S. Korea/2023	Lee S et al. (2023)
MP beads	101 Beads	2850, 2950, 3010	-	Germany/2023	Glockler et al. (2023)
Optical fibers	310 µm diameter	853, 925, 1081, 1264, 1648, 1736	C–C–O str O–CH_3_ rock C–C skeletal, str C–COO str (C–H) of α-CH_3_ (C–H) of O–CH_3_	India/2008	Thomas et al. (2008)
Polydisperse sample	Single 10 µm particle	-	-	France/2021	Elsayed et al. (2021)
Common consumer products	< 5000	960, 980	C = O str	USA/2023	Sipps et al. (2023)
Seafood	< 1.6	-	-	France/2016	Dehaut et al. (2016)
MP samples	-	602, 736, 811	-	China/2023	Ren et al. (2023)
Contaminated tap water	15–150 Microbead	3002	-	Germany/2019	Kniggendorf et al. (2019)
Landfill leachate and sludge	Majority less than 50	-	-	China/2021	Sun et al. (2021)
River water	Mean 2.12 ± 0.02	-	-	Spain/2023	Trujillo et al. (2023)
Airborne MPs collected from indoor environments	2.5–336.89 fibers, foams, pellets, fragments, films, lines	-	-	Turkey/2024	Bhat et al. (2024a)
Bleached mangrove (environmental) MP samples	< 5 mm	-	-	China/2023	Liu Y et al. (2023)
Beach sediment	< 400	-	-	Germany/2016	Kappler et al. (2016)
Aqueous solution	3.4 microspheres	414, 574, 748, 596, 812, 970, 990, 1450, 1725, 1640	-	Austria/2020	Derksen et al. (2020)

### Polyurethane

The Raman spectral profile of PU is presented in Table 18. PU is a highly versatile, durable polymer known for its chemical and abrasion resistance. It consists of repeating carbamic (urethane) moieties (Feldman 2008; França de Sá *et. al.* 2016; Howard et al*.* (Howard, 2002); Zia et al. 2007). Developed in 1937 by Dr. Otto Bayer, PU is synthesized through xenobiotic processes involving the condensation of polyalcohols and polyisocyanates, which confer its chemical, thermal, and mechanical tunability (Howard et al*.* 2022; Zia et al. 2007). Depending on the linearity of its structure, PU can vary from soft and flexible to hard and rigid. PU is widely used in the automotive industry, adhesives, fibers, elastomers, binding agents, construction materials, thermoplastics, synthetic leathers, furniture, paints, coatings, and biomedical applications. The majority of PU produced is used in foamed plastics (Feldman 2008; Howard et al*.* 2022; Zia et al. 2007). In Raman spectroscopy, PU is identified by the presence of the urethane (–NH–CO–O–) group. Figure S15 displays the Raman spectrum of PU MPs. Characteristic peaks include C–N stretching with amide III bands at 1220–1300 cm⁻^1^, amide II bands associated with N–H bending at 1530 cm⁻^1^ (Kumar et al. 2021; Saad et al. 2022), C = O stretching of the carbonyl group at 1730 cm⁻^1^, and CH₂ stretching vibrations at 2940–2970 cm⁻^1^ (Saad et al. 2022).

**Table 18:** Raman spectral profile of PU MPs

Source	Size (µm)	Raman fingerprint (cmˉ^1^)	Country/year	Ref
Marine sediment	40–1000 Fiber, sphere, fragment	-	USA/2022	Cashman et al. (2022)
Drinking water plants	1–50	1650, 2850	Germany/2023	Maurizi et al. (2023)
Marine environment	> 12	-	France/2024	Grand et al. (2024)
Mussel samples	3–60 Fibers	1170, 1450, 1600	Germany/2021	Kumar et al. (2021)
River/fresh water	0.5–5 mm Fiber, fragment	1250, 1620, 2910	S. Africa/2022	Saad et al. (2022)
Marine and estuarine sediments	45–1000 fragment	-	USA/2023	El Khatib et al. (2023)
Seafood	< 1.6	-	France/2016	Dehaut et al. (2016)
Bleached MP samples	213.12 particle	-	China/2023	Liu Y et al. (2023)

### Acrylonitrile butadiene styrene

Table 19 presents the Raman spectral profile of ABS, a rigid and durable engineering plastic. ABS is typically synthesized through additive manufacturing polymerization processes and is known for its exceptional toughness, dimensional stability, low thermal expansion coefficient, and resistance to impact, chemicals, and cold temperatures. It also offers ease of cost-effective processing. Originally developed during World War II for molding bulletproof sheets, profiles, and pipes, ABS has since found widespread use in products such as LEGO toys, home appliances, textiles, and 3D printing. ABS is generally formed by polymerizing monomers into a matrix of acrylonitrile-styrene (AS), with butadiene particles evenly distributed throughout the matrix. By altering the morphological and molecular structure, such as the type, size, and quantity of butadiene particles, the physical properties of ABS can be significantly modified (Lenz et al. 2015). ABS MPs have been detected as fibers and fragments in root vegetables, aerosols, Icelandic sediments, and marine organisms using Raman spectroscopy (Kazour et al. 2019; Loughlin et al. 2021; Ren et al. 2023; Tympa et al. 2021; Yoo et al. 2023). Figure S16 displays the Raman spectrum of ABS. Its characteristic peaks resemble those of PS, including benzene ring deformation at 621 cm⁻^1^, benzene ring breathing at 1002 cm⁻^1^, in-plane C–H deformation of the benzene ring at 1032 cm⁻^1^, benzene ring stretching at 1603 cm⁻^1^, and C–H stretching of the benzene ring at 3060 cm⁻^1^. A distinguishing feature of ABS, not present in PS, is the characteristic C≡N stretching peak at 2239 cm⁻^1^ (Tympa et al. 2021).

**Table 19:** Raman spectral profile of ABS MPs

Source	Size (µm)	Raman fingerprint (cmˉ^1^)	Band assignment	Country/year	Ref
Root vegetables	0.3–100 Powder	620 1000 1602 3058	Ring of benzene Benzene ring breathing C–C vibration ring = C-H of benzene ring Str	Greece/2021	Tympa et al. (2021)
PM10 aerosol	> 10	600, 1000, 3060, 2235	Characteristic CN peak	S. Korea/2023	Yoo et al. (2023)
MP samples	-	619 1004 1453 1605	Benzene ring vibr C–C benzene ring vibr C–C str CH str	China/2023	Ren et al. (2023)
Iceland sediments	> 150 Fibers	-	-	Ireland/2021	Loughlin et al. (2021)
Mussels	< 200 Fibers, fragments	-	-	France/2019	Kazour et al. (2019)
MP samples	-	619, 1004, 1453, 1605	-	China/2023	Ren et al. (2023)
MP samples	300–1000	1004, 1155, 1453, 1605, 2240, 2913, 3055	-	China/2024	Liu et al. (2024)
Vegetal (moss) matrices	0.8–65.4	-	-	France/2023	Hagelskjaer et al. (2023)
Human placenta	1.03–6.84	-	-	China/2024	Yun et al. (2024)
Common consumer products	< 5000	-	-	USA/2023	Sipps et al. (2023)
Atlantic Ocean	≥ 10 particles	1001 2236 2900–3060	Aromatic ring C☰N stretch C-H stretch	Denmark/2015	Lenz et al. (2015)

### Polyacrylonitrile

Table 20 presents the Raman spectral profile of PAN, a synthetic fiber widely used in industry due to its excellent mechanical strength, thermal stability, chemical resistance, and overall durability (Feldman 2008). PAN microfibers have been detected in marine environments and within the tissues of marine organisms. Similar to other textile-based polymers, PAN poses environmental risks to living organisms upon release into aquatic ecosystems (Genchi et al. 2023; Kumar et al. 2021; Laptenok et al. 2020). A characteristic Raman spectral band of PAN MPs is the strong –C≡N stretching vibration observed at 2289 cm⁻^1^ (Dong et al. 2014). Figure S17 illustrates the Raman spectrum of PAN MPs (Weingrill et al. 2023).

**Table 20:** Raman spectral profile of PAN MPs

Source	Size (µm)	Raman fingerprint (cmˉ^1^)	Country/year	Ref
Fish GIT	10–20 Fiber	2245 2954	S. Arabia/2020	Laptenok et al. (2020)
Marine environment	170–410 fiber	2200 2850	S. Arabia/2023	Genchi et al. (2023)
Mussel samples	3–60 Fiber, fragments	1600, 2700	Germany/2020	Kumar et al. (2021)
Fish	0.22–9.5 mm fibers	-	Belgium/2017	Collard et al. (2017)
Human placentas	1–17 (average of ~ 6.24)	450, 972, 1317, 1458, 2871, 2940, 3591	US/2023	Weingrill et al. (2023)

## Raman spectrum results of other common MP types

In addition to the Raman features of the previously discussed MPs, the spectra of several other environmentally detected MPs are summarized in Table 21.

**Table 21:** Raman spectral profile of other common MP types

Source	Size (µm)	Raman fingerprint (cmˉ^1^)	Band assignment	Country/year	Ref
Polyethylene/hexene-1-copolymer (PEH)					
River/fresh water	0.5–5 mm Fiber, fragment	1050, 1295, 1450, 2900	-	S. Africa/2022	Saad et al. (2022)
Polybutyl acrylate (PBA)					
Drinking water plant	1–50	2950	-	Germany/2023	Maurizi et al. (2023)
Polybutylene terephthalate (PBT)					
WWTP	25–250 Fibers	-	-	Czech Republic/2023	Spacilova et al. (2023)
MP samples	-	633, 707, 859, 1108, 1288, 1615, 1721	-	China/2023	Ren et al. (2023)
MP samples	300–1000	633, 1108, 1288, 1615, 1721, 2815, 3036	-	China/2024	Liu et al. (2024)
Polyvinyl alcohol (PVOH, PVA)					
Fish and mussel	5.4–15.1 Fiber, fragment, film	1270, 1420, 1650, 2800–3000	-	Greece/2023	Miserli et al. (2023)
Human breastmilk	2–12 Irregular fragments	650, 1150, 1200, 1500, 1520	-	Italy/2022	Ragusa et al. (2022)
Landfill leachate and sludge	Majority less than 50	-	-	China/2021	Sun et al. (2021)
Marine sediments	> 0.45	-	-	New Zealand/2023	Ribo et al. (2023)
River water	Mean 2.12 ± 0.02	-	-	Spain/2023	Trujillo et al. (2023)
Pyrolysized sewage sludge	Fragments, films, and fibers, mostly under 200	-	-	China/2020	Ni et al. (2020)
Airborne lab MPs	1–1000 fibers	-	-	UK/2024	Bhat et al. (2024b)
Polyvinylidene fluoride (PVDF)					
Stormwater drainage systems	25–530 Fragments, fibers	813, 2990	-	Australia/2022	Bond et al. (2022)
Thin films	13–218 nm	412.0, 536.3, 611.7, 796.1, 839.9, 874.4	-	Romania/2024	Purica et al. (2024)
Poly (*n*-butyl methacrylate) (PBMA)					
Fish	5.4 Film, fragment, fibers	1648 3100	ν(C = C) and ν(C–COO) modes (C = O) of C–COO	Greece/2023	Miserli et al. (2023)
Landfill leachate and sludge	Majority less than 50	-	-	China/2021	Sun et al. (2021)
Fish	0.22–9.5 mm fragments	-	-	Belgium/2017	Collard et al. (2017)
Thin polymer films	Nanoparticles	~ 1730, ~ 2900	C = O C-H	Poland/2024	Khaksar et al. (2024)
Marine sediments	> 0.45	-	-	New Zealand/2023	Ribo et al. (2023)
Pyrolysized sewage sludge	Fragments, films, and fibers, mostly under 200	-	-	China/2020	Ni et al. (2020)
Polystyrene acrylonitrile (PSA) (acrylic)					
Deep ocean	> 100 < 5	-	-	USA/2019	Choy et al. (2019)
Poly (ethylene–vinyl acetate) PEVA					
Fish and mussels	5.4–15.1 Film, fragments, fibers	1150, 1400, 1550, 2820, 2950–3000	-	Greece/2023	Miserli et al. (2023)
River/fresh water	0.5–5 mm Fiber, fragment	1120, 1300, 1450, 2850, 2900	-	S. Africa/2022	Saad et al. (2022)
Lake samples	20–80	-	-	China/2023	Ma et al. (2023)
Landfill leachate and sludge	Majority less than 50	-	-	China/2021	Sun et al. (2021)
Polyvinyl-butyral (PVB)					
Fish and mussels	5.4–15.1 Fiber, films, fragments	1250–1350 1420–1500 1720 2850–3000	C–O–C str CH₂ bending C = O str -CH,—CH₂ str	Greece/2023	Miserli et al. (2023)
Landfill leachate and sludge	Majority less than 50	-	-	China/2021	Sun et al. (2021)
Marine sediments	> 0.45	-	-	New Zealand/2023	Ribo et al. (2023)
Polyoxymethylene (POM)					
Wastewater sludge	Particle fibers	-	-	Finland/2021	Asamoah et al. (2021)
MP samples	-	602, 811	-	China/2023	Ren et al. (2023)
MP samples	300–1000	811, 1332, 1486, 2913, 2995	-	China/2024	Liu et al. (2024)
Airborne MPs collected from indoor environments	2.5–336.89 sized fibers, foams, pellets fragments, films, lines	-	-	Turkey/2024	Bhat et al. (2024)
Bleached MP samples	152.83 particle	-	-	China/2023	Liu Y et al. (2023)
Poly (ethylene-co-acrylic acid) (EAA)					
Mussels and seawater	15.1 1–5 mm	1050, 1300, 1450, 2850, 2900	-	Greece/2023	Miserli et al. (2023)
Pyrolysized sewage sludge	Fragments, films, and fibers, mostly under 200	-	-	China/2020	Ni et al. (2020)
White polycarbonate (WPC)					
MP samples	-	750, 900, 1290, 1600	-	Belgium/2024	Plazas et al. (2024)
Melamine resin (MR)					
MPs + natural organic matter	2 Sphere	976, 2960	-	S. Korea/2023	Lee S et al. (2023)
Polyacrylamide (PAM)					
Fish brain	8–96 Fragment, film	1650 2850	-	Portugal/2023	Barboza et al. (2023)
Landfill leachate and sludge	Majority less than 50	-	-	China/2021	Sun et al. (2021)
Airborne lab MPs	1–1000 fibers	-	-	UK/2024	Bhat et al. (2024)
Acrylic acid					
Marine sediments	> 0.45	-	-	New Zealand/2023	Ribo et al. (2023)
Polyacrylic acid (PAA)					
Fish brain	8–96 Fragment, film	1450, 2850	-	Portugal/2023	Barboza et al. (2023)
Polyethylene glycol/oxide (PEG/PEO)					
Airborne MPs collected from indoor environments	2.5–336.89 sized fibers, foams, pellet fragments, films, lines	-	-	Turkey/2024	Bhat et al. (2024)
Polyvinyl stearate (PVS)					
DWTP	> 20	-	-	Italy/2024	Barchiesi et al. (2024)
Polylactic acid (PLA)					
Coffee lid	250–1000 nm	-	-	Germany/2023	Meyns et al. (2023)
MP samples	300–1000	1101, 1345, 1468, 1943	-	China/2024	Liu et al. (2024)
River water	Mean 2.12 ± 0.02	-	-	Spain/2023	Trujillo et al. (2023)
Polycaprolactone (PCL)					
MP samples	300–1000	2646, 2925	-	China/2024	Liu et al. (2024)
Bleached MP samples	134.62 particle	-	-	China/2023	Liu Y et al. (2023)
Thermoplastic urethane (TPU)					
MP samples	300–1000	1185, 1248, 1306, 1440, 1605, 1740, 2925	-	China/2024	Liu et al. (2024)
Plastic starch materials (PSM)					
MP samples	300–1000	1456, 2846, 2879, 2943	-	China/2024	Liu et al. (2024)
Ethylene vinyl acetate copolymer (EVA)					
MP samples	300–1000	1440, 2849, 2884	-	China/2024	Liu et al. (2024)
Vegetal (moss) matrices	0.8–65.4	-	-	France/2023	Hagelskjaer et al. (2023)
Sewage sludge	Fragments, films, and fibers, mostly under 200	-	-	China/2020	Ni et al. (2020)
Airborne MPs collected from indoor environments	2.5–336.89 sized fibers, foams, pellets fragments, films, lines	-	-	Turkey/2024	Bhat et al. (2024)
Atlantic Ocean	≥ 10 μm particles	444, 610, 2850	-	Denmark/2015	Lenz et al. (2015)
Polymethyl pentene (PMP)					
Landfill leachate and sludge	Majority less than 50	-	-	China/2021	Sun et al. (2021)
Airborne lab MPs	1–1000 fibers	-	-	UK/2024	Bhat et al. (2024)
Polyaryletherketone (PAEK)					
Landfill leachate and sludge	Majority less than 50	-	-	China/2021	Sun et al. (2021)
Polybutadiene acrylonitrile (PBAN)					
Airborne MPs collected from indoor environments	2.5–336.89 sized fibers, foams, pellets fragments, films, lines	-	-	Turkey/2024	Bhat et al. (2024)
Polyhydroxyalkanoates (PHA)					
Bleached MP samples	104.23 particle	-	-	China/2023	Liu Y et al. (2023)
Polybutylene sebacate (PBS)					
Bleached MP samples	213.12 particle	-	-	China/2023	Liu Y et al. (2023)

## Advancements, standardization, and limitations

### Advancements

Raman spectroscopy has become an important analytical technique for identifying and characterizing MPs across environmental and biological matrices. Its principal advantages, rapid and often non-destructive analysis, minimal sample preparation, and high spatial resolution, make it exceptionally suitable for diverse environmental applications (Ribeiro-Claro et al. 2017). Table 22 summarizes diagnostic Raman bands of common polymers frequently detected in MPs. A notable advantage of Raman spectroscopy is its weak sensitivity to water interference, which allows accurate detection of MPs even in aqueous samples. In contrast to FT-IR spectroscopy, whose broad water bands can mask polymer peaks, Raman spectra remain well-resolved due to the low polarizability of water molecules (Ribeiro-Claro et al. 2017). This enables the identification of particles as small as 1 μm, well below the ≈ 20 μm resolution typically achievable by FT-IR (Araujo et al. 2018).

**Table 22:** Diagnostic Raman shifts of common polymers

Polymer	Raman shifts (cmˉ^1^)		Assignments	Ref
Polyethylene		~ 1060 ~ 1126 ~ 1294 ~ 1365 ~ 1415, 1438 ~ 1463 ~ 2846 ~ 2875–2881	C–C asymmetric stretching C–C symmetric stretching CH₂ twist/rocking at ~ 1300 cm⁻^1^ CH₂ wagging CH₂ bending CH₂ rocking CH₂ symmetric stretching CH₂ asymmetric stretching	Sunil et al. (2024a) Kadac-Czapska et al. (2023)
Polyethylene terephthalate		~ 628 ~ 701 ~ 790 ~ 856 ~ 1114 ~ 1175 ~ 1289 ~ 1411 ~ 1613 ~ 1727 ~ 2800 and 3000 ~ 3090	C–C–C in-plane bending C–C–C out of plane bending C–H out of plane bending C–C breathing mode C–H in-plane bending C–H in-plane bending, C–C Stretching C(O)–O stretching CCH bending, OCH bending Phenyl C–C ring stretching C = O stretching CH₂ stretching vibrations Phenyl C–H stretch	Kadac-Czapska et al. (2023) Sunil et al. (2024a)
Polypropylene Polyamide		~ 397 ~ 807 ~ 839 ~ 971 ~ 1035 ~ 1150 ~ 1217 ~ 1327 ~ 1458 ~ 2811 ~ 2885 ~ 2954–2963 ~ 940 ~ 1085, 1106, 1126 ~ 1294, 1307 ~ 1440 ~ 1463 ~ 2882 ~ 1634 ~ 1270	CH₂ wagging, CH bending CH₂ rock, C–C and C–CH₃ str C–C stretching, CH₃ rocking C–C stretching, CH₃ rocking C–CH₃ str., C–C str C–C stretching, CH₃ rocking C–C stretching, CH₃ bending CH stretching, CH₃ bending, CH₂ wagging CH₂ bending, CH₃ asymmetric bending CH₂ stretching CH₃ symmetric stretching CH₃ asymmetric stretching C–CO stretching C–C stretching CH₂ twisting vibrations CH₂ bending CH₂ rocking C–H stretching C = O (amide I band) C–N stretching and N–H bending vibrations	Perraki et al. (2024) Sunil et al. (2024a) Kadac-Czapska (2023) Sunil et al. (2024a)
Polystyrene Polyvinyl chloride		~ 621 ~ 793 ~ 1000 ~ 1030 ~ 1153 ~ 1582 ~ 1600 ~ 2900 and > 3000 ~ 361 ~ 636 ~ 695 ~ 1179 ~ 1431 ~ 2914	C–C-C ring deformation mode C–C-C ring deformation mode Benzene ring stretching mode C–H in-plane deformation C–H in-plane bending C = C stretch C = C stretch/aromatic C–H aliphatic/aromatic) C–Cl in trans HClC = CHCl in-plane bending C–Cl in Cl₂C = CHCl stretching mode C–Cl in H₂C = CHCl asymmetric stretching CH₂ twisting CH₂ bending CH₂ bending	Sunil et al. (2024a) Xue et al. (2022) Liu et al. (2022) Sunil et al. (2024a)
Polycarbonate		~ 576, 637, 826 ~ 705, 735 ~ 887 ~ 1110 ~ 1179, 1236 ~ 1461 ~ 1604 ~ 2800–3100	Benzene ring vibration C–H bending O–C(O)–O stretching C–H in-plane bending C–O–C stretching CH₃ deformation Benzene ring stretch C–H stretching modes	Sunil et al. 2024a Xue et al. 2022
Polymethyl methacrylate		~ 600 ~ 810 ~ 968 ~ 1449 ~ 1727	C–C–O stretching C–O–C symmetric stretching C–C stretching C–H bending C = O stretching	Lee S et al. (2023)
Polydimethylsiloxane		~ 1254 ~ 1417–1435 ~ 2899	Asymmetric stretching vibrations of Si–CH₃ groups Asymmetric bending deformation modes of CH₃ groups C–H stretching modes of methyl groups bonded to silicon atoms	Durdakvova et al. (2020)
Polyester		~ 701 ~ 857 ~ 1284 ~ 1461 ~ 1613 ~ 1726	C–C ring stretching C–O–C bending COO stretching CH₂ bending C = C stretching C = O stretching	Loppi et al. (2021)

Technological innovations have considerably advanced Raman’s analytical power. Confocal Raman microscopy, Raman–atomic-force microscopy (Raman-AFM), CARS, and SERS now enable detailed 2D and 3D chemical imaging of MPs in biological tissues, sediments, and soils (Galluzzi et al. 2025; Xu et al. 2020; Yin et al. 2021; Yun et al. 2024). CRT further accelerates data acquisition, achieving up to 100 × faster imaging than conventional Raman setups (Grand et al. 2024).

The incorporation of artificial-intelligence and machine-learning algorithms, such as principal-component analysis/linear-discriminant analysis (PCA-LDA) and artificial neural networks (ANNs), has improved automation and spectral interpretation (Qi et al. 2023; Sunil et al. 2024b). Portable and handheld Raman spectrometers now enable in situ MP/NP detection without extensive laboratory preparation (Farale et al. 2025). Although current portable units remain less sensitive than benchtop systems, their potential for rapid field deployment is promising. Collectively, these advances confirm Raman spectroscopy’s growing role as a primary diagnostic tool for MP research and environmental monitoring.

### Spectral libraries and computational tools

Table 23 summarizes the major Raman spectral libraries and computational tools currently employed for the identification and characterization of MPs. These resources collectively illustrate the growing integration of digital spectroscopy, open-access data sharing, and ML in modern MP analysis.

**Table 23:** Spectral libraries and computational tools for identification of MPs

Tool/library	Description	Key features	Polymers covered	Accessibility	Ref
SLoPP/SLoPP-E	Open-access Raman libraries for MPs (including weathered variants)	148 + 113 spectra; morphology and color diversity; high hit-quality match when combined with commercial libraries American Chemical Society Publications + 2PubMed + 2	PE, PP, PS, PET, PVC, PA, PMMA, PU	Free download; compatible with Bio-Rad KnowItAll	Munno et al. (2020)
KnowItAll Raman	Commercial database with MP add-ons	> 27,000 spectra; automated preprocessing; large multi-material library Wiley Science Solutions + 2Wiley Science Solutions + 2	Core synthetic polymers; some additives/aging variants	Subscription required	Wiley Science Solutions (2025)
RamanSPy	Open-source Python toolkit for Raman workflows	Baseline and cosmic-ray correction; modular preprocessing and analysis; user-defined polymer classes	User-defined (e.g., PE, PET, PS)	Free via GitHub	Georgiev et al. (2024)
RamanNet	Machine-learning toolkit for Raman MP data	CNN-based noise handling; ML classification workflows; demonstrated high accuracy on benchmark data	PE, PP, PET, PS, PVC	Free Python package	Ibtehaz et al. (2023)
ML-Raman (MPs)	ML identifier for MP Raman spectra	Random-forest or CNN classification; > 90% accuracy on test matrices	PE, PP, PET, PS, PVC	Free/opensource (arXiv)	Ramanna et al. (2023)

The SLoPP and SLoPP-E spectral libraries (Munno et al. 2020) represent widely utilized open-access databases that provide extensive reference spectra for both pristine and environmentally weathered polymers. Their inclusion of diverse morphologies and colors enhances the accuracy of spectral matching, particularly when used in conjunction with commercial databases such as KnowItAll Raman (Wiley Science Solutions 2025), which offers automated preprocessing and an extensive spectral repository. However, the subscription-based model of KnowItAll limits accessibility for some research institutions.

Recent advances in computational spectroscopy have introduced robust open-source frameworks, including RamanSPy (Georgiev et al. 2024), which provides comprehensive functionalities for baseline correction, cosmic-ray removal, multivariate analysis, and data visualization. Similarly, ML-based platforms such as RamanNet (Ibtehaz et al. 2023) and ML-Raman (Ramanna et al. 2023) have achieved high classification accuracy for common polymer types across complex matrices. These ML-driven tools enhance analytical throughput and reproducibility while reducing reliance on expert interpretation and proprietary libraries.

Collectively, the resources summarized in Table 23 highlight an emerging trend toward standardization, openness, and computational automation in Raman-based MP characterization. The increasing availability of curated spectral libraries and ML-enabled open-source platforms is expected to accelerate method harmonization, enhance data interoperability, and promote reproducible MP identification across laboratories and environmental contexts.

### Standardization

The advancement of Raman-based MP analysis now depends on establishing robust international standards encompassing harmonized methods, certified reference materials (CRMs), and interlaboratory validation. Three strategic priorities are proposed: ring trials, certified materials, and metadata-based reporting frameworks.

Interlaboratory ring trials are essential for benchmarking reproducibility and quantifying analytical bias. Global comparisons indicate overall detection accuracies of 50–70% for MPs < 50 μm, mainly due to inconsistent preprocessing (Ciornii et al. 2025). Expanding such trials through coordinated networks, such as the Network of Reference Laboratories for Monitoring Emerging Environmental Pollutants (NORMAN) or Global Water Research Coalition (GWRC), could improve recovery rates to > 90% across laboratories (Zhang et al. 2024).

The establishment of CRMs underpins analytical traceability. The European Commission’s European Reference Material (EURM)-060 (released 2025) provides PET-based particles (1–1000 μm) dispersed in water for method validation and limit-of-detection assessment (Jacob et al. 2024; Stefaniak et al. 2025).

Table 24 outlines a proposed framework for Raman standardization, emphasizing QA/QC, spectral preprocessing, and data transparency. Harmonized protocols, including consistent baseline correction, smoothing, and normalization, are necessary for reproducible analysis (Qi et al. 2023). Implementing a minimum-metadata schema (laser wavelength, acquisition parameters, reference library, and matrix type) will enhance data interoperability across studies (Cowger et al. 2020; Zhang et al. 2024).

**Table 24:** Proposed framework for Raman standardization

Standardization element	Objective	Recommended practice	Expected outcome	Ref
Interlaboratory trials	Evaluate reproducibility	Conduct annual blind tests with shared MP standards	± 10% agreement in ID and counts	Ciornii et al. (2025)
Certified reference materials	Calibration and validation	Use PET-based	Harmonized Raman response curves	Jacob et al. (2024); Stefaniak et al. (2025)
Quality assurance	Minimize contamination	Use natural-fiber apparel, DI-rinsed glassware, and procedural blanks	Reduced false positives	Ribeiro-Claro et al. (2017)
Data preprocessing standards	Ensure spectral comparability	Apply baseline correction, normalization, and smoothing protocols	Improved signal-to-noise ratio	Qi et al. (2023)
Metadata reporting	Enhance transparency	Include laser type, acquisition parameters, and spectral libraries	Reproducible data sharing	Zhang et al. (2024)
Open spectral libraries	Facilitate cross-study matching	Deposit verified spectra to public repositories	Global interoperability	Grand et al. (2024)
Training and certification	Build analytical capacity	Implement operator certification for Raman–MP workflows	Improved inter-lab reliability	Sunil et al. (2024b)

Finally, analyst training and certification should accompany procedural harmonization to ensure consistent data acquisition and interpretation. Combined with open-access spectral repositories, these measures will transform Raman spectroscopy from a primarily research-level technique into a standardized, regulatory-ready method for global MP monitoring.

### Limitations of this review

This review synthesizes Raman spectral data and advancements from 207 studies on MP detection, but inherent challenges in the field and literature gaps impose constraints. Transparent acknowledgment of these limitations highlights avenues for future enhancements.

### Scope and selection bias

Focus on prevalent polymers (PS, PET, PE, PP) and matrices (water, sediment, air, biological tissues) aligns with dominant literature but overlooks emerging types (bio-based polylactic acid) and underrepresented contexts (deep soils, non-Western studies). This may bias toward well-studied systems, limiting breadth for niche applications like agricultural runoff.

### Heterogeneous data quality

Spectra from varied sources (532–1064 nm lasers, inconsistent preprocessing) introduce inconsistencies in profiles, exacerbated by incomplete reporting on weathering shifts. Without raw data, meta-analyses were infeasible, potentially underestimating spectral variability. Proposed metadata schemas (Table 24) could address this.

### Quantitative and temporal gaps

Emphasis on qualitative signatures limits depth on metrics like detection limits (1–10 µm in water vs. 50–200 µm in sediments) or recovery rates (> 90% with KOH). Current through 2025 (for example, CRT; Grand et al. 2024), it risks obsolescence amid rapid AI evolution (for instance, CNNs; Ren et al. 2023). Economic barriers to techniques like SERS/CARS are underexplored, hindering applicability in low-resource settings.

These constraints position the review as a foundational resource, not exhaustive. They underscore priorities for global databases, interlaboratory trials, and inclusive methods to foster equitable MP research.

## Supplementary Information

Below is the link to the electronic supplementary material.

Supplementary file1 (DOCX 1.32 MB)

## Data Availability

This review article synthesizes data from previously published studies, and no new primary data were generated. All data supporting the findings, including Raman spectral profiles and sample preparation protocols, are derived from the literature cited in the references. Figures are reproduced with permission from their respective sources. For access to the original data, please refer to the cited publications.
