# Supplementary material for: Applications of Raman spectroscopy for microplastic detection and characterization: a comprehensive spectral reference
Source: Environ Sci Pollut Res Int. 2025 Nov 27;32(50):28630–77. doi: 10.1007/s11356-025-37224-3 (PMC12714846; doi:10.1007/s11356-025-37224-3)
Supplement: Supplementary file 1 — Supplementary file1 (DOCX 1.32 MB) [file 11356_2025_37224_MOESM1_ESM.docx]

**Supporting Information**


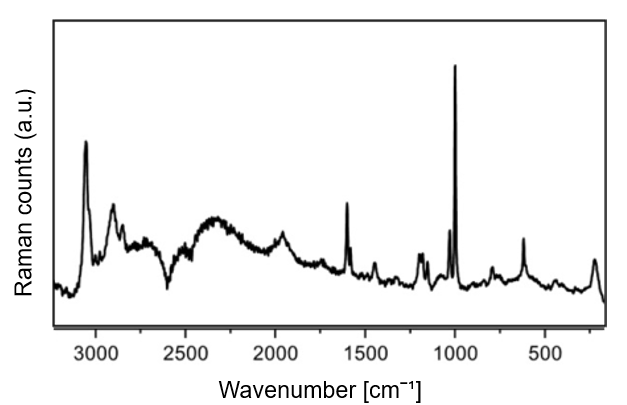


**Figure S1**. Raman spectrum of polystyrene (PS) microplastics. Reprinted with permission from Rytelewska and Dabrowska 2022.


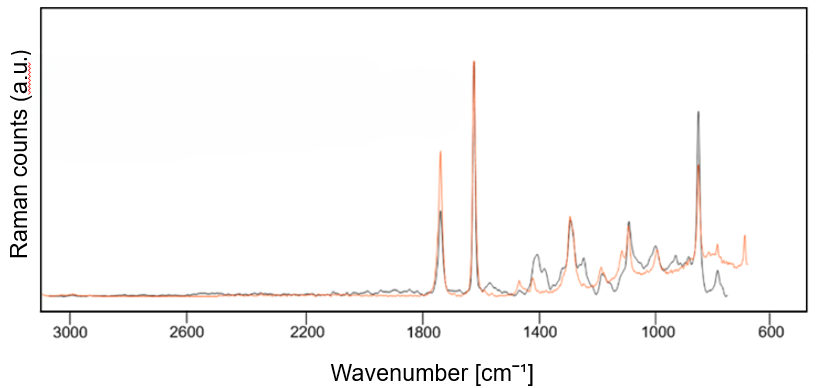


**Figure S2**. Raman spectrum of polyester (PES) microplastics. Reprinted with permission from Thiele *et al*. 2023.


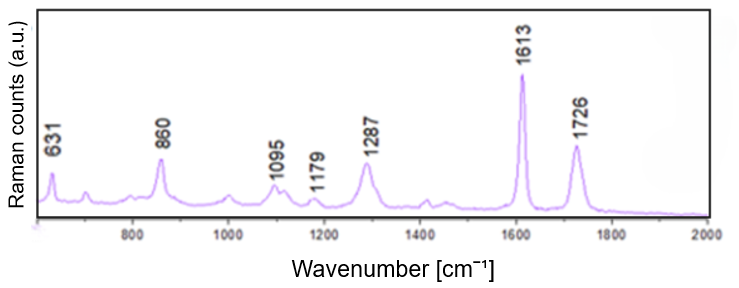


**Figure S3**. Raman spectrum of polyethylene terephthalate (PET) microplastics. Reprinted with permission from Manolaki *et al*. 2023.


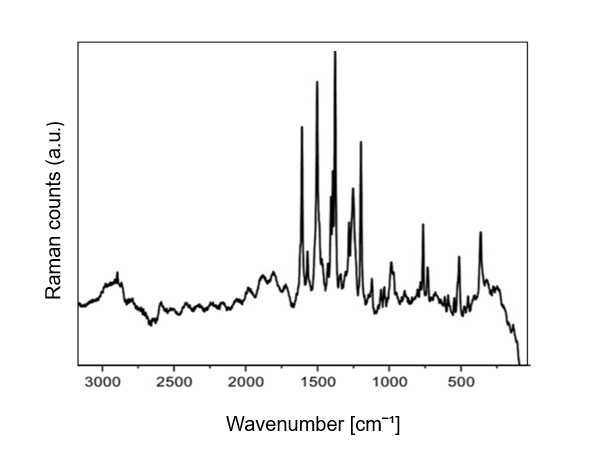


**Figure S4**. Raman spectrum of polyamide (PA) microplastics. Reprinted with permission from Rytelewska and Dabrowska 2022.


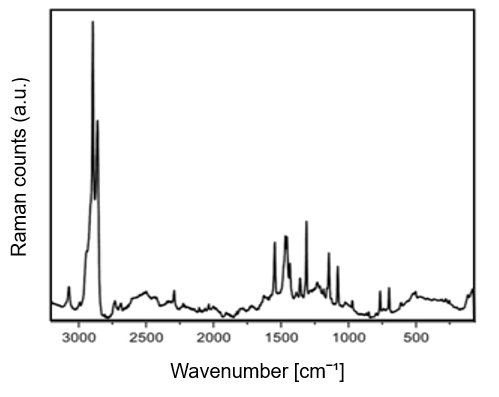


**Figure S5**. Raman spectrum of polyethylene (PE) microplastics. Reprinted with permission from Rytelewska and Dabrowska 2022.


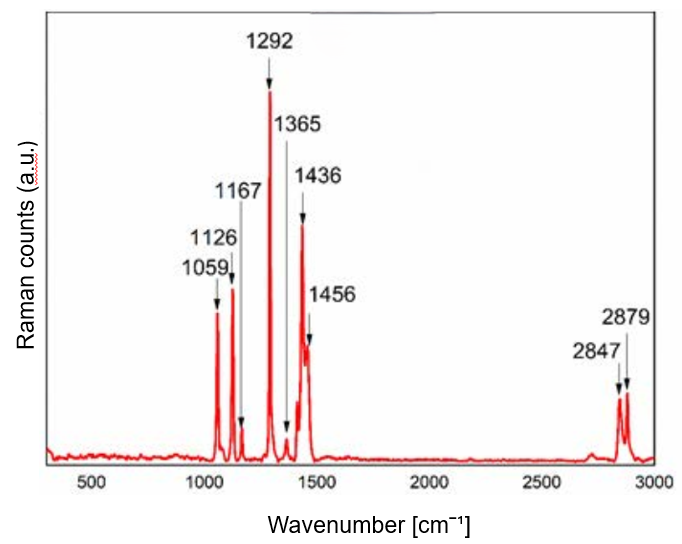


**Figure S6**. Raman spectrum of high-density polyethylene (HDPE) microplastics. Reprinted with permission from Unnimaya *et al.* 2023.


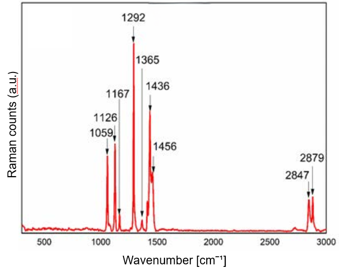


**Figure S7**. Raman spectrum of low-density polyethylene (LDPE) microplastics. Reprinted with permission from Unnimaya *et al.* 2023.


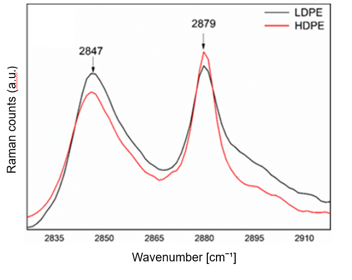


**Figure S8.** Comparison of Raman spectra in the CH₂ stretching frequency region, showing peaks at 2879 cm⁻¹ and 2847 cm⁻¹ for HDPE and LDPE. Reprinted with permission from Unnimaya *et al.* 2023.


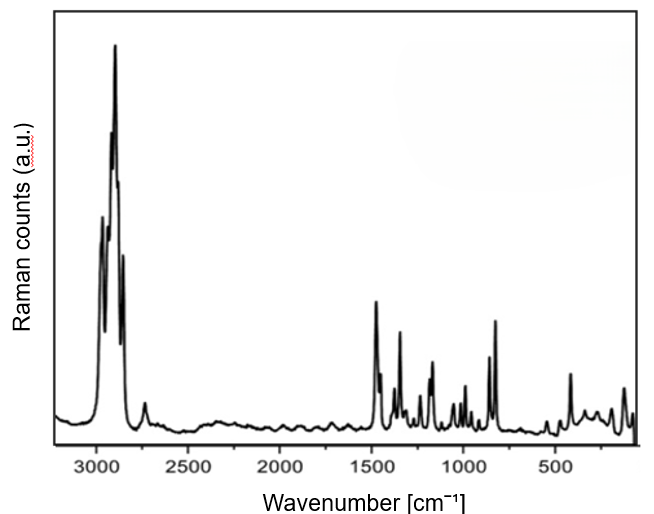


**Figure S9**. Raman spectrum of polypropylene (PP) microplastics. Reprinted with permission from Rytelewska and Dabrowska 2022.


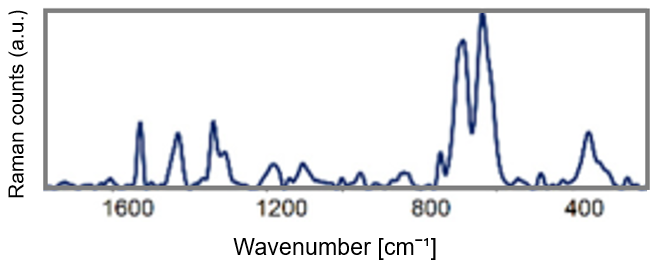


**Figure** S**10**. Raman spectrum of polyvinyl chloride (PVC) microplastics. Reprinted with permission from Ragusa *et al*. 2022.


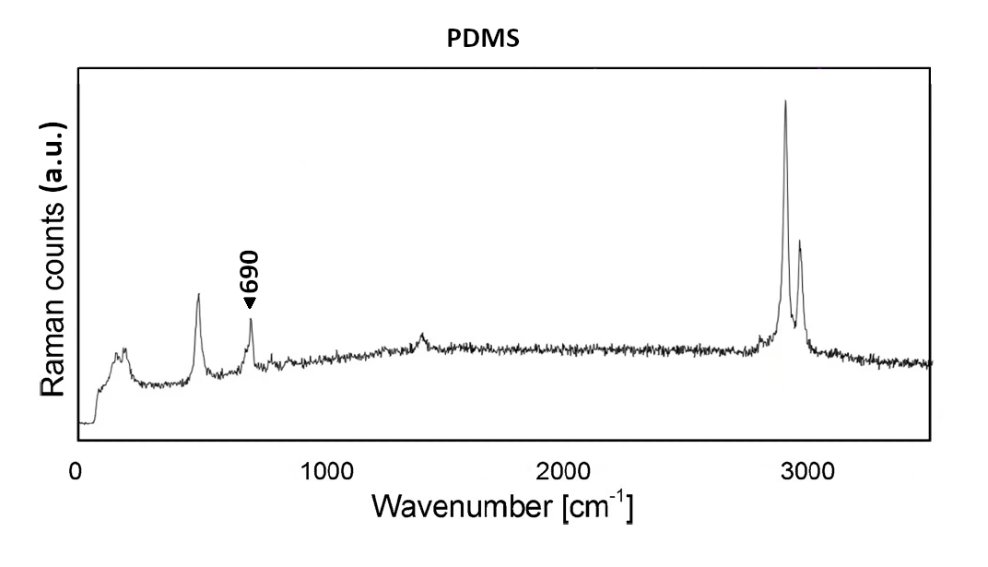


**Figure S11.** Raman spectrum of polydimethylsiloxane (PDMS) microplastics. Reprinted with permission from Kernchen *et al*. 2024.


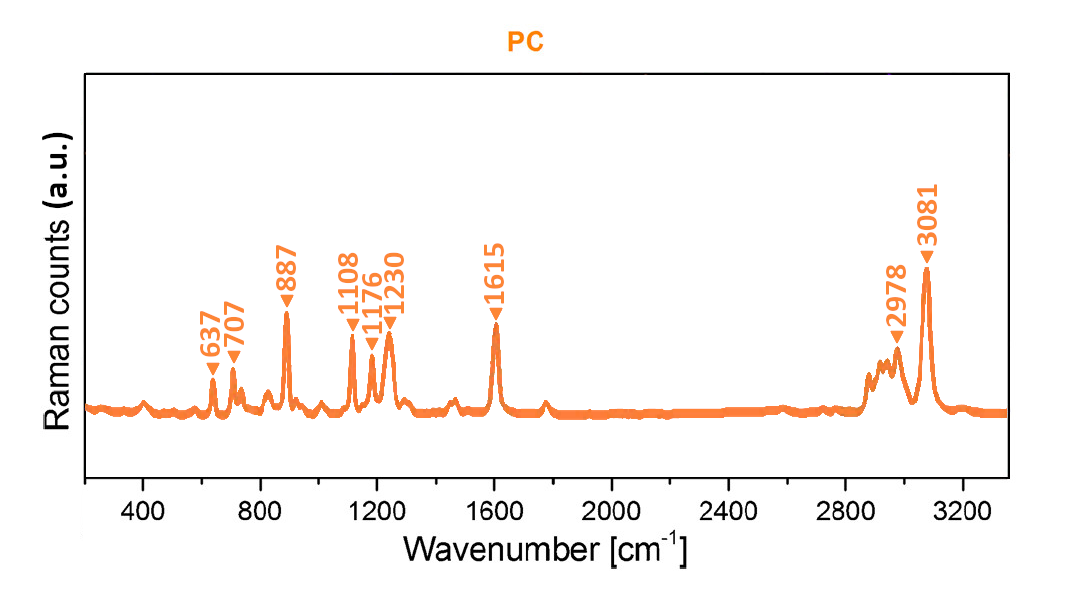


**Figure S12.** Raman spectrum of polycarbonate (PC) microplastics. Peak positions were adapted from Jin *et al*. 2022 and Liu *et al*. 2024. Reprinted with permission from Kappler *et al*. 2016.


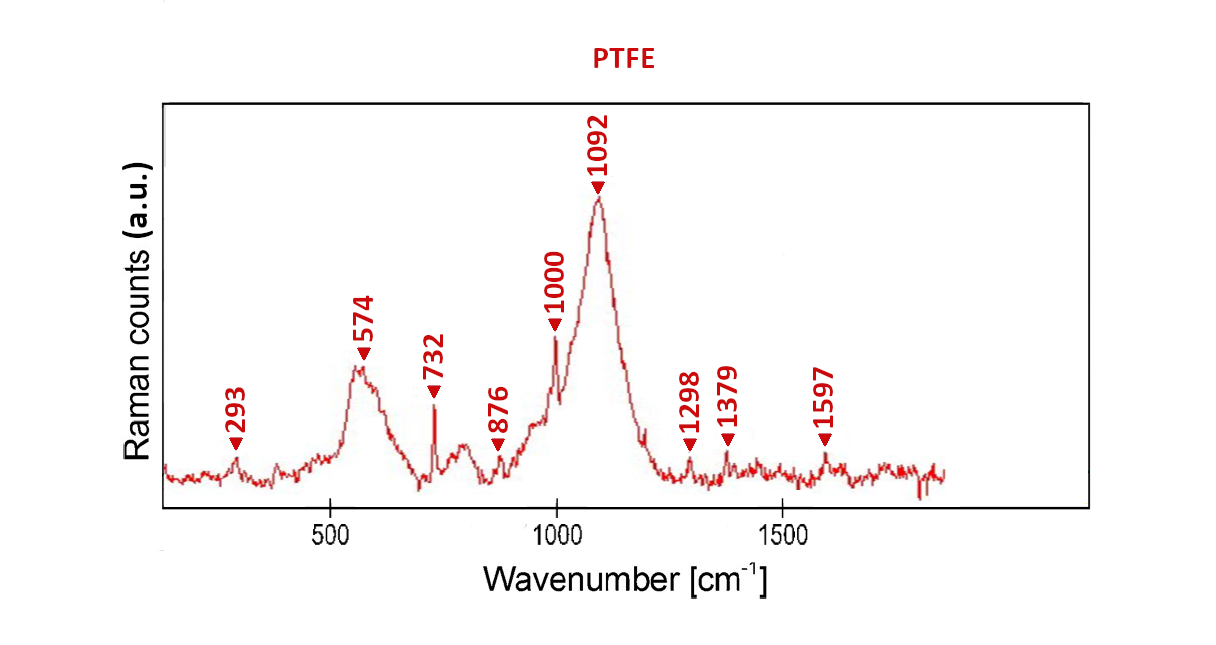


**Figure S13**. Raman spectrum of polytetrafluoroethylene (PTFE) microplastics. Peaks positions were adapted from Pu *et al*. 2025. Reprinted with permission from Karpenko *et al*. 2021.


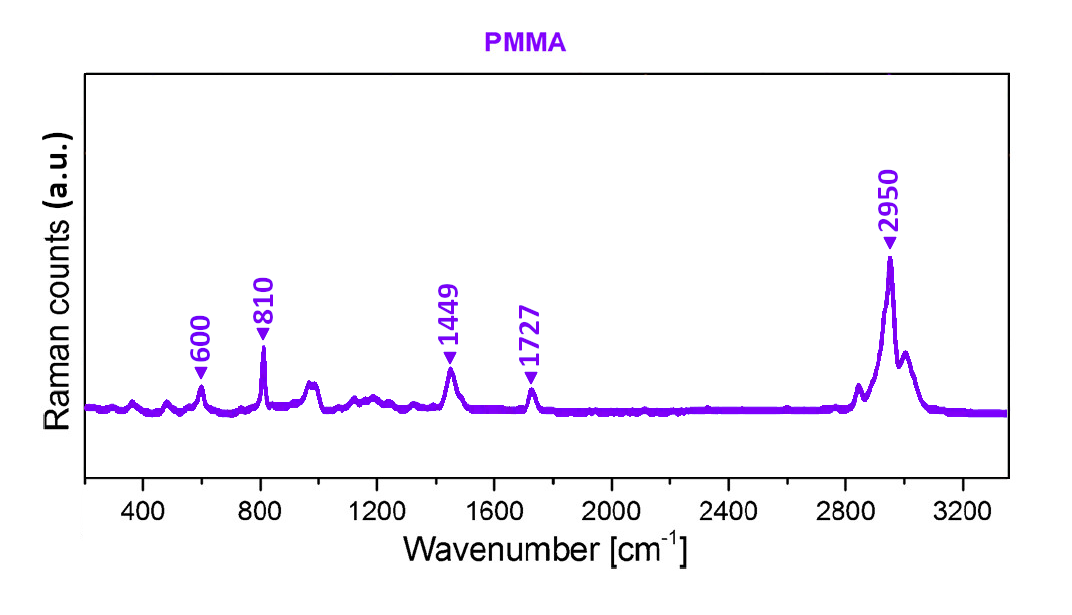


**Figure S14**. Raman spectrum of polymethylmethacrylate (PMMA) microplastics. Reprinted with permission from Kappler *et al*. 2016.


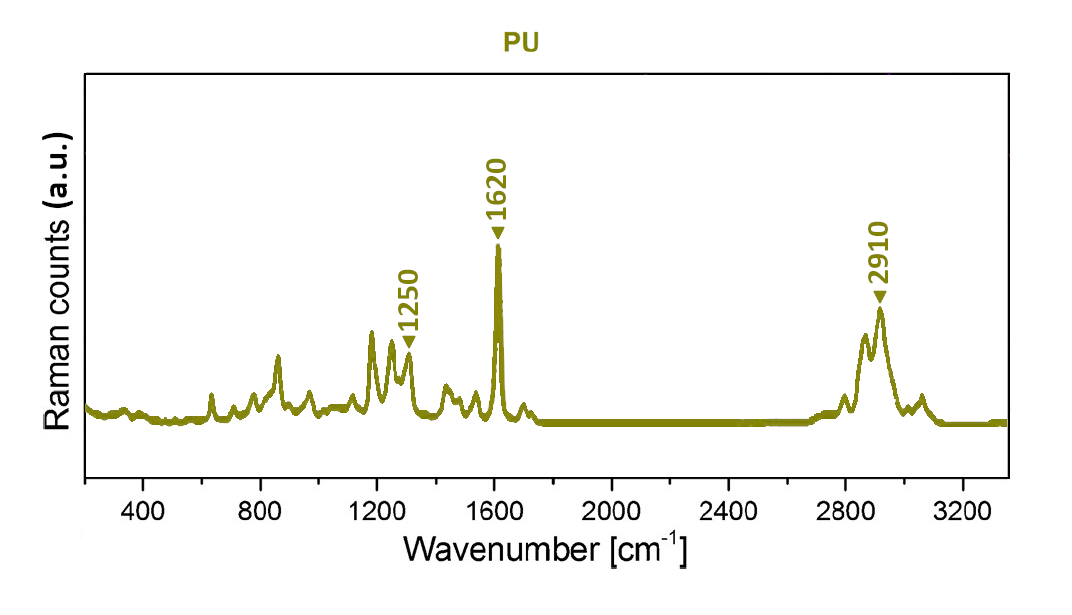


**Figure S15**. Raman spectrum of polyurethane (PU) microplastics. Reprinted with permission from Kappler *et al*. 2016.


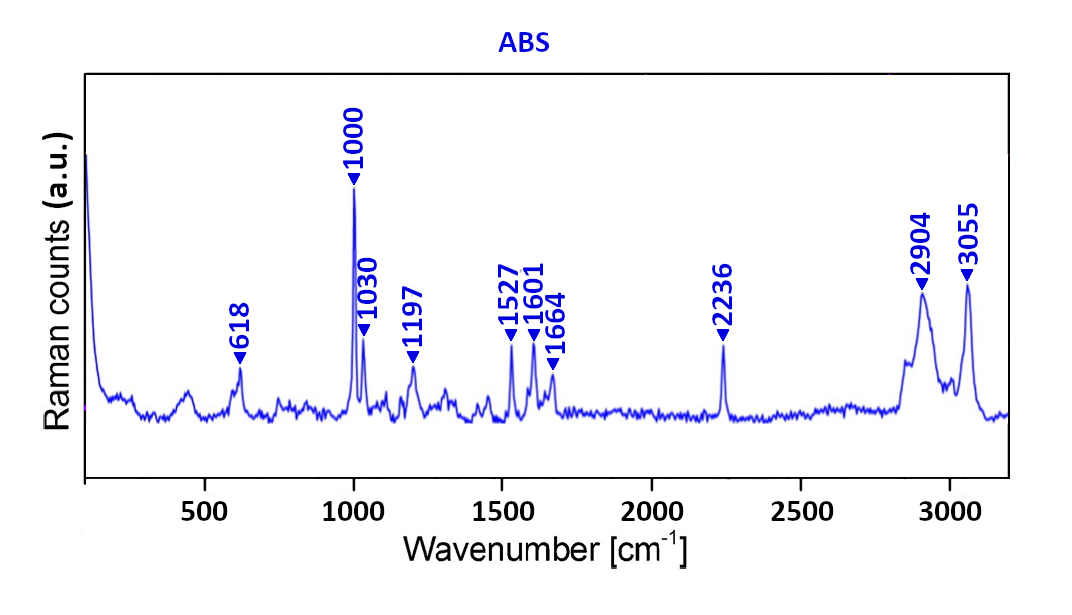


**Figure S16**. Raman spectrum of acrylonitrile butadiene styrene (ABS) microplastics. Reprinted with permission from Lenz *et al*. 2015.


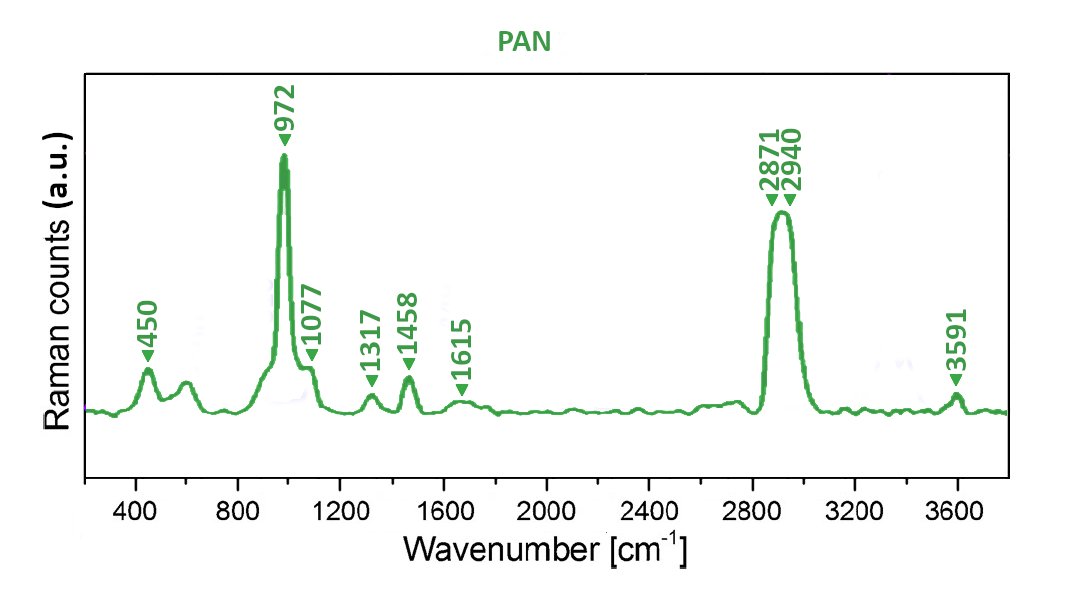


**Figure S17**. Raman spectrum of polyacrylonitrile (PAN) microplastics. Reprinted with permission from Weingrill *et al*. 2023.
